# Cancer‐Associated Fibroblast‐Derived Sphingosine‐1‐Phosphate Activates a MALL–SDC4 Axis to Facilitate Perineural Invasion in Pancreatic Cancer

**DOI:** 10.1002/advs.75426

**Published:** 2026-04-22

**Authors:** Wang Peng, Mengdie Cao, Hai Huang, Shuya Bai, Luyao Liu, Jingwen Liang, Haochen Cui, Qiaodan Zhou, Shiru Chen, Jiamei Jiang, Luoxia Liu, Zhou Luan, Wei Chen, Si Xiong, Ronghua Wang, Bin Cheng, Yuchong Zhao

**Affiliations:** ^1^ Department of Gastroenterology and Hepatology Tongji Hospital Tongji Medical College Huazhong University of Science and Technology Wuhan China; ^2^ Department of Nuclear Medicine Tongji Hospital Tongji Medical College Huazhong University of Science and Technology Wuhan China; ^3^ Department of Gastroenterology Shandong Provincial Hospital Affiliated to Shandong First Medical University Jinan China; ^4^ Department of Internal Medicine University of Pittsburgh Medical Center Mercy Hospital Pittsburgh Pennsylvania USA

**Keywords:** cancer‐associated fibroblasts, MAL‐like protein, pancreatic ductal adenocarcinoma, perineural invasion, Schwann cells, sphingosine‐1‐phosphate, tumor microenvironment

## Abstract

Pancreatic ductal adenocarcinoma (PDAC) frequently exhibits perineural invasion (PNI), a clinicopathologic feature strongly associated with local recurrence and poor survival, yet lacking effective targeted interventions. By integrating patient cohorts with single‐cell and bulk transcriptomics, multiplex immunofluorescence, and functional assays, this study defines a stromal‐tumor signaling axis facilitating neural invasion. Cancer‐associated fibroblasts (CAFs), particularly a myofibroblastic CAF‐enriched population, upregulate sphingosine kinase 1 (SPHK1) and increase secretion of sphingosine‐1‐phosphate (S1P), which activates sphingosine‐1‐phosphate receptor 3 (S1PR3)/JNK/JUN signaling to transcriptionally induce MAL‐like protein (MALL) in cancer cells. MALL binds to syndecan‐4 (SDC4) and promotes its recycling to the plasma membrane, thereby increasing surface SDC4 abundance. This MALL–SDC4 program promotes RhoA/phosphorylated myosin light chain 2 (p‐MLC2)‐dependent amoeboid motility and sensitizes cancer cells to Schwann cell‐derived pleiotrophin, strengthening directed neural invasion. Disruption of the axis through SPHK1 knockdown in CAFs, genetic perturbation of MALL or SDC4 in cancer cells, or adeno‐associated virus‐mediated SPHK1 or SDC4 knockdown in KPC (*Kras* ^LSL‐G12D/+^; *Trp53* ^LSL‐R172H/+^; *Pdx1*‐Cre) mice significantly reduces PNI and tumor burden. These findings uncover a metabolite‐driven MALL–SDC4 program connecting stromal metabolism to neural invasion, and identify promising therapeutic targets for PDAC.

## Graphical abstract

CAF‐derived S1P activates the S1PR3‐JNK‐Jun signaling pathway, thereby transcriptionally upregulating MALL expression in cancer cells. MALL increases the cell‐surface abundance of SDC4 by promoting its recycling and restricting its lysosomal degradation, which in turn enhances RhoA‐p‐MLC2‐dependent amoeboid invasion and SDC4‐PTN axis‐mediated cancer cell‐Schwann cell communication, ultimately promoting perineural invasion.

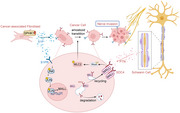

## 1 Introduction

Pancreatic ductal adenocarcinoma (PDAC) represents one of the most lethal malignancies worldwide, with a dismal 5‐year survival rate of less than 13%, and it is projected to become the second leading cause of cancer‐related death by 2040 [1, 2]. Among its aggressive features, perineural invasion (PNI), defined as the presence of cancer cells along nerves or within the epineural, perineural, and endoneurial spaces, occurs in approximately 70–98% of cases, making it the solid tumor with the highest PNI incidence, and represents a critical driver of disease progression [3]. PNI is strongly associated with local recurrence, refractory pain, and reduced survival [4]. Although the complex tumor microenvironment (TME) is recognized as a critical regulator of PNI, the cellular and molecular mechanisms underlying this neural tropism remain incompletely defined.

The TME of PDAC is a complex ecosystem, characterized by a dense desmoplastic stroma containing diverse non‐malignant cells that actively support tumor progression [5]. In PNI, interactions between cancer cells and stromal components are critical. Cancer‐associated fibroblasts (CAFs), the predominant stromal cell population in PDAC, are well‐established promoters of tumor growth, metastasis, and therapeutic resistance. However, their specific role in initiating PNI remains poorly understood [6, 7]. Growing evidence indicates that CAFs undergo metabolic reprogramming and support tumor growth through the secretion of nutrients and metabolites such as acetate and lipids [8, 9, 10]. Recent studies indicate that, beyond providing metabolic fuel, CAF‐derived metabolites can act as signaling molecules that directly modulate cancer cell behavior [11, 12, 13]. Schwann cells, the principal glial cells of the peripheral nervous system, have emerged as key mediators of tumor‐nerve crosstalk. Through secretion of chemokines, neurotrophic factors, and extracellular matrix components, they establish a permissive niche that facilitates cancer cell invasion along nerves [14, 15, 16]. Although the individual contributions of CAFs and Schwann cells to PNI are increasingly recognized, their cooperative mechanisms remain enigmatic. This knowledge gap is especially critical in PDAC, which exhibits intense desmoplasia and the highest incidence of PNI among solid tumors. Despite growing interest, previous studies have largely examined these stromal components in isolation, and understanding their potential synergy in forming a pro‐invasive niche remains a core challenge in deciphering the mechanism of PNI.

Recent evidence revealed that cancer cells employ highly adaptive strategies to invade the dense and confining perineural space. A key mechanism is their transition to amoeboid migration—a rapid, protease‐independent movement driven by RhoA–ROCK–myosin II contractility, which provides a critical advantage during perineural invasion [17, 18]. The amoeboid phenotype features dynamic control of cell shape and cell size, enabling cancer cells to assume a rounded, deformed morphology capable of squeezing through narrow perineural clefts without degrading the matrix. These characteristics are particularly advantageous for cancer cells to deform and squeeze through tight stromal matrices, given that prominent desmoplasia and extensive stromal components comprise up to 60–90% of the tumor mass in PDAC [4, 8, 19]. However, the specific signals within the tumor microenvironment that trigger this amoeboid transition during perineural invasion remain unidentified. Moreover, the molecular mechanisms that mediate communication between migrating cancer cells and neural structures to complete the invasion process have yet to be fully elucidated.

In this study, we show that CAFs elevate sphingosine kinase 1 (SPHK1) and secrete the bioactive lipid metabolite sphingosine‐1‐phosphate (S1P) in the PNI niche, which transcriptionally upregulates MAL‐like protein (MALL) in cancer cells. MALL stabilizes syndecan‐4 (SDC4) to promote an amoeboid phenotype, enhancing both RhoA‐driven motility and cancer cell responsiveness to pleiotrophin (PTN) derived from Schwann cells. This intercellular signaling network couples CAF metabolism to Schwann cell‐fueled neural invasion, and identifies therapeutic vulnerabilities for targeting PNI in PDAC.

## 2 Results

### 2.1 Cancer‐Associated Fibroblasts are Enriched in PDAC with Perineural Invasion and Clinically Cooperate with Schwann Cells

**FIGURE 1:**
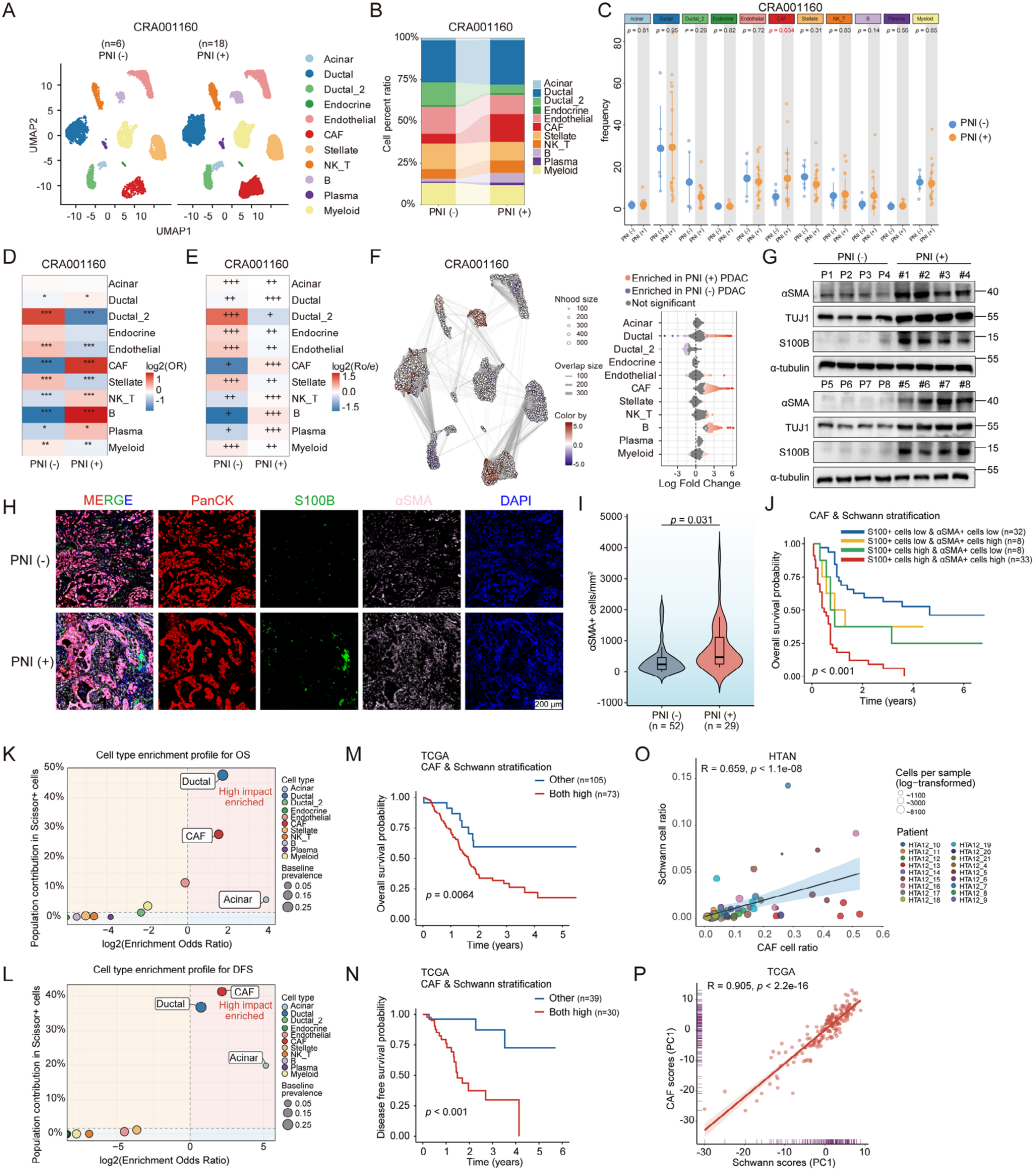
Cancer‐associated fibroblasts are enriched in PDAC with perineural invasion and clinically cooperate with Schwann cells. A) UMAP visualization of the PDAC scRNA‐seq dataset CRA001160 showing distinct cell populations with PNI clinical annotations (PNI(‐): n = 6; PNI(+): n = 18). B) Percentage stacked bar chart displaying cell type compositions stratified by PNI status. C) Boxplot showing cell type frequencies according to PNI status. D) Heatmap of odds ratios (OR) prioritizing CAFs as a major differential population according to PNI status. E) Heatmap of observed‐to‐expected ratios (Ro/e) prioritizing CAFs as a major differential population according to PNI status. F) MiloR neighborhood differential abundance analysis according to PNI status. The left panel shows a UMAP plot of cellular neighborhoods; the right panel displays a beeswarm plot of neighborhood‐level differences summarized by cell type. G) Western blot analysis demonstrating expression levels of cell type‐specific markers for neurons (TUJ1), Schwann cells (S100B), and CAFs (αSMA) in PDAC tumor samples. H) Representative multiplex immunofluorescence images demonstrating higher CAF abundance in PNI‐positive tumors compared with PNI‐negative tumors. Scale bars, 200 µm. I) Quantification of αSMA‐positive CAF density stratified by PNI status in a PDAC tissue microarray cohort (n = 81). J) Kaplan‐Meier survival curves for overall survival stratified by the combined αSMA‐positive CAF and S100B‐positive Schwann cell abundance in the tissue microarray cohort (n = 81). K, L) Scatter plots visualizing the association of cell types with OS (K) and DFS (L) evaluated by Scissor analysis. The y‐axis represents the proportion of Scissor‐positive cells; the x‐axis shows the enrichment odds ratio. M, N) Kaplan‐Meier survival curves for OS (M) and DFS (N) stratified by combined CAF and Schwann cell scores in the TCGA‐PAAD dataset. O) Positive correlation between CAF and Schwann cell ratios at the single‐cell level in the PDAC scRNA‐seq dataset HTAN (60 samples from 18 patients). P) Positive correlation between CAF and Schwann cell scores in the TCGA‐PAAD dataset, confirming the cooperative relationship between these cell types. Correlation analyses were performed using the Spearman correlation method. Survival curves were analyzed using the log‐rank test. Data are presented as mean (SD) and were analyzed using Student's *t*‐test for two‐group comparisons or one‐way ANOVA followed by Tukey's post‐hoc test for multiple comparisons as appropriate.

To identify cell populations associated with PNI, we performed cell preference analysis and compared cellular compositions between PNI‐positive (n = 18) and PNI‐negative (n = 6) tumors in the single‐cell RNA sequencing (scRNA‐seq) dataset CRA001160 (Table S1). Cell type annotation with canonical marker expression revealed distinct populations including ductal cells, acinar cells, endocrine cells, immune cells, endothelial cells, stellate cells, and CAFs (Figure 1A, Figure S1A). Cell ratio analysis revealed a shift in cellular composition, with CAFs constituting a substantially enlarged proportion of the tumor microenvironment in PNI‐positive samples (Figure 1B). Cell frequency analysis further demonstrated that CAFs were significantly more abundant in PNI‐positive than in PNI‐negative tumors (Figure 1C). Complementary approaches consistently validated this enrichment. Odds ratio (OR) analysis indicated a strong association between CAF presence and PNI status, while observed‐to‐expected ratio (Ro/e) analysis confirmed their marked overrepresentation in PNI cases (Figure 1D,E). Furthermore, MiloR's neighborhood differential abundance analysis robustly identified CAFs as a major differentially abundant cell population linked to PNI status (Figure 1F). Given the marked accumulation of CAFs in PNI‐positive tumors, we next examined CAF heterogeneity. We performed AddModuleScore‐based subtype analysis using established myofibroblastic CAF (myCAF), inflammatory CAF (iCAF), and antigen‐presenting CAF (apCAF) signatures in the CRA001160 dataset. This analysis identified three CAF clusters, among which cluster 0 showed the highest myCAF signature scores, cluster 2 showed the highest iCAF signature scores, and cluster 1 did not show strong enrichment for any of the three canonical signatures (Figure S1B,C). We then found that myCAF‐like cells (cluster 0) were preferentially enriched in the PNI‐positive group by proportion, odds ratio, and observed‐to‐expected analyses (Figure S1D–F), indicating that the PNI‐associated stromal expansion is driven by specific CAF states rather than a uniform increase across all subtypes.

PNI is fundamentally defined by the invasion of cancer cells into the neural compartment. Schwann cells constitute the critical cellular interface and structural docking site that cancer cells must engage to invade nerves. Western blot analysis confirmed the concurrent upregulation of cell markers for CAFs, neurons, and Schwann cells in PNI‐positive tumor samples (Figure 1G). Immunofluorescence staining showed that Schwann cells (marked by S100B) co‐localized with NF200‐positive axonal structures and TUJ1‐positive neuronal elements along peripheral nerve fibers within the PDAC microenvironment (Figure S1G). Consequently, we sought to determine whether CAFs cooperate with Schwann cells in PDAC. Multiplex immunofluorescence analysis revealed that PNI‐positive tumors exhibited elevated CAF abundance (Figure 1H,I, Figure S1H). Consistently, Kaplan‐Meier survival analysis demonstrated that tumors with dual‐high levels of S100B‐positive cells and αSMA‐positive cells were associated with worse overall survival (Figure 1J). We further performed Scissor analysis, which identifies cell populations associated with clinical outcomes [20]. Scatter plots revealed that CAFs were prominently positioned in the upper right quadrant with both a high proportion within Scissor‐positive cells and an elevated enrichment odds ratio for poor overall survival (OS) and disease‐free survival (DFS) outcomes, indicating CAFs as pivotal contributors to adverse clinical outcomes in PDAC (Figure 1K,L, Figure S1I,J). Due to the relative scarcity of Schwann cells in pancreatic tumors and technical limitations of single‐cell sequencing, most PDAC scRNA‐seq datasets, including the aforementioned CRA001160, lacked a sufficient number of annotated Schwann cells. To overcome this limitation, we identified the Human Tumor Atlas Network (HTAN) dataset, where we successfully annotated 3365 Schwann cells across 60 samples from 18 patients (Figure S1K,L; Table S1). Since only one patient in this cohort was PNI‐negative, this dataset was excluded from the cell preference analysis; additionally, samples within the dataset that failed to capture Schwann cells were also excluded from this study. We then used this dataset to generate CAF and Schwann cell signatures, and calculate signature scores (defined as the first principal component, PC1; see Supporting Information: “Survival and Scissor Analysis”) in The Cancer Genome Atlas pancreatic adenocarcinoma cohort (TCGA‐PAAD). The combined signature score stratification revealed a synergistic prognostic effect. Tumors with dual high scores exhibited the poorest OS and DFS in the TCGA‐PAAD dataset (Figure 1M,N). Remarkably, we observed a strong positive correlation between CAF and Schwann cell ratios at the single‐cell level in the HTAN dataset (Figure 1O). The TCGA‐PAAD dataset further validated this correlation (Figure 1P). Spatial transcriptomics analysis using the Cell2location deconvolution algorithm revealed that regions with high Schwann cell abundance were co‐enriched with CAFs, providing spatial evidence for their potential cooperation in promoting neural invasion (Figure S1M).

The predominant accumulation of CAFs in PNI is particularly critical in PDAC, a malignancy defined by its extensive desmoplastic stroma. Growing evidence indicates that stromal cells, including CAFs, play essential roles in promoting neural invasion [15]. Consistent enrichment patterns observed across multiple analytical approaches strongly suggest that CAFs actively remodel the microenvironment to facilitate neural invasion. Importantly, these CAFs appear to cooperate with Schwann cells to drive the perineural invasion process.

### 2.2 Cancer‐Associated Fibroblast‐Secreted Metabolites Facilitate Perineural Invasion

**FIGURE 2:**
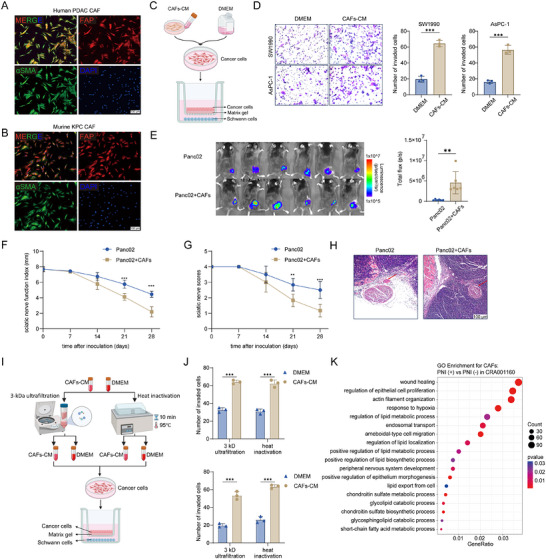
Cancer‐associated fibroblast‐secreted metabolites facilitate perineural invasion. A, B) Immunofluorescence characterization of human PDAC‐derived (A) and KPC‐derived (B) primary cancer‐associated fibroblasts showing positive expression of αSMA and FAP with characteristic spindle‐shaped morphology. Scale bars, 100 µm. C) Schematic diagram of the transwell co‐culture model: Schwann cells seeded in the lower chamber with cancer cells pretreated for 24 h with either DMEM control or CAF‐conditioned medium placed in the upper chamber. D) Quantification demonstrating that CAF‐CM pretreatment significantly increases cancer cell invasion toward Schwann cells compared with DMEM control. E) In vivo sciatic nerve invasion model showing elevated bioluminescence imaging signal intensity in mice co‐injected with cancer cells and CAFs compared with cancer cell‐only controls (n = 6 per group). F, G) Co‐injection of cancer cells with CAFs significantly exacerbates nerve dysfunction as evaluated by sciatic nerve function index (F) and limb function scores (G). H) Hematoxylin and eosin staining images showing extensive neural encasement in CAF co‐injected mice compared with controls. Scale bars, 100 µm. I) Workflow for conditioned medium fractionation, including protein heat inactivation (95°C, 10 min) and 3‐kDa molecular weight cutoff ultrafiltration to isolate small molecules. J) Functional validation showing that filtered and heat‐inactivated CAF‐CM retains pro‐invasion activity compared with DMEM and filtered/heat‐inactivated DMEM controls, implicating small metabolites as active factors. K) Gene Ontology functional enrichment analyses comparing CAFs from PNI‐positive and PNI‐negative cases in the PDAC scRNA‐seq dataset CRA001160. Data are presented as mean (SD) and were analyzed using Student's *t*‐test for two‐group comparisons or one‐way ANOVA followed by Tukey's post‐hoc test for multiple comparisons as appropriate. ^*^ *p* < 0.05, ^**^ *p* < 0.01, ^***^ *p* < 0.001.

To elucidate the functional role of CAFs in PNI, we isolated primary CAFs from human PDAC tumors and murine KPC (*Kras^LSL‐G12D/+^; Trp53^LSL‐R172H/+^; Pdx1‐Cre*) tumors, and confirmed their identity through immunofluorescence (Figure 2A,B). We designed a Transwell co‐culture model to simulate cancer cell invasion toward Schwann cells. Using this system, we observed that CAF‐conditioned medium (CM) significantly enhanced cancer cell invasion toward Schwann cells relative to control medium (Figure 2C,D). These findings suggest that CAF‐derived soluble factors facilitate perineural invasion.

In vivo validation using a sciatic nerve invasion model demonstrated that co‐injection of cancer cells with CAFs led to increased bioluminescence signals and aggravated nerve dysfunction, as measured by sciatic nerve function index and limb function scores (Figure 2E–G). Gross examination and histological assessment further revealed enhanced tumor growth and extensive neural encasement in mice receiving CAF co‐injection (Figure 2H, Figure S2A). These results confirmed the pro‐invasive role of CAFs in vivo.

To identify the active invasion‐promoting components within CAF‐CM, we performed biochemical fractionation experiments using heat inactivation of proteins and ultrafiltration with a 3‐kDa molecular weight cutoff (Figure 2I). Notably, both heat‐inactivated and filtered CAF‐CM retained significant pro‐invasion activity compared with control treatments (Figure 2J, Figure S2B), suggesting that small metabolites, rather than proteins, serve as the key mediators. Gene Ontology (GO) analysis comparing PNI‐positive versus PNI‐negative groups revealed distinct pathway alterations in cancer cells and CAFs separately. For cancer cells, PNI‐positive tumors showed significant pathway activation of amoeboid‐type cell migration and cellular response to nutrient levels (Figure S2C). This finding is consistent with recent evidence that cancer cells utilize amoeboid migration to facilitate invasion through dense stromal environments during perineural invasion [18]. Importantly, this cell‐type‐specific enrichment analysis using single‐cell data provides biologically relevant insights that reflect the genuine functional states of distinct cellular populations within the tumor microenvironment. For CAFs, PNI‐positive cases demonstrated pronounced activation of metabolic pathways compared with PNI‐negative counterparts (Figure 2K). Subsequent analysis of laser capture microdissection RNA sequencing data from three independent datasets revealed upregulation of 27 metabolic enzymes in stromal regions (including CAFs, immune cells, and endothelial cells) compared with tumor areas (Figure S2D). To identify the cellular origins of these stroma‐upregulated metabolic enzymes, we performed cell marker analysis in the CRA001160 scRNA‐seq dataset. The feature plot visualized the number of metabolic enzymes upregulated in each cell type, revealing that CAFs exhibited the highest degree of enrichment compared with other cell populations (Figure S2E). Complementary analysis using AddModuleScore for all 27 metabolic enzymes demonstrated the highest scores in CAFs compared with other cell types (Figure S2F). These results further establish CAFs as the principal source of heightened metabolic activity in the PDAC stroma. Collectively, these results directed our focus toward CAF‐derived metabolites as potential key drivers of the observed pro‐invasive phenotype in PNI.

### 2.3 Cancer‐Associated Fibroblasts Secrete Sphingosine‐1‐Phosphate to Promote Perineural Invasion

**FIGURE 3:**
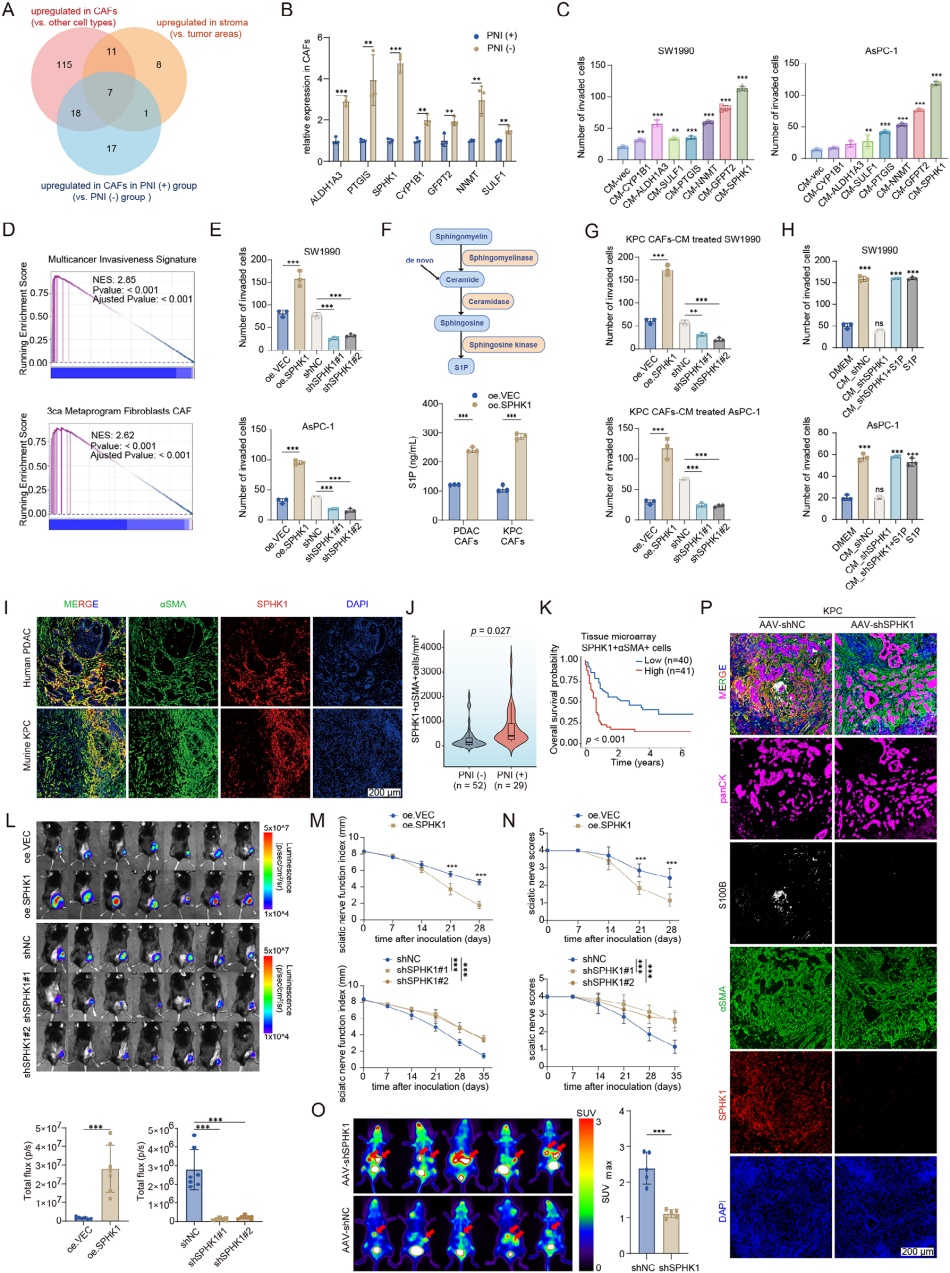
Cancer‐associated fibroblasts secrete sphingosine‐1‐phosphate to promote perineural invasion. A) Venn plot showing the number of overlapping enzymes. B) qPCR validation confirming upregulation of the seven candidate metabolic enzymes in CAFs from PNI‐positive versus PNI‐negative tumors. C) Functional screening of conditioned media from HEK293T cells overexpressing each candidate enzyme to identify the primary pro‐invasion driver through Transwell invasion assays. D) Gene Set Enrichment Analysis of TCGA‐PAAD tumors with high SPHK1 expression. E) Functional validation showing that modulating SPHK1 expression in primary human CAFs significantly alters their conditioned medium's pro‐invasion effect on cancer cells. F) Sphingolipid metabolism pathway diagram and ELISA quantification demonstrating increased S1P levels in conditioned medium from SPHK1‐overexpressing human and mouse CAFs compared with controls. G) Functional validation showing that modulating SPHK1 expression in KPC‐derived primary CAFs significantly alters their conditioned medium's pro‐invasion effect on cancer cells. H) Functional assay showing that exogenous S1P treatment (5 µm) significantly enhances cancer cell invasion toward Schwann cells. I) Immunofluorescence images demonstrating the SPHK1 expression in CAFs in both human and murine pancreatic tumors. Scale bars, 200 µm. J) Quantification of SPHK1+αSMA+ cell density stratified by PNI status in a PDAC tissue microarray cohort (n = 81). K) Kaplan‐Meier survival curves for overall survival stratified by SPHK1+αSMA+ cell abundance in the PDAC tissue microarray cohort (n = 81). L) Bioluminescence images showing that modulating SPHK1 expression in primary KPC CAFs significantly alters tumor burden measured by bioluminescence signal intensity in the sciatic nerve invasion mouse model (n = 7 per group). M, N) Functional assessment showing that modulating SPHK1 expression in primary KPC CAFs significantly alters sciatic nerve function index (M) and limb scores (N) in the sciatic nerve invasion mouse model. O) Positron emission tomography/computed tomography (PET/CT) imaging showing that AAV‐mediated SPHK1 knockdown significantly reduces tumor burden as evaluated by maximum standardized uptake value (SUVmax) in KPC mice (n = 5 per group). P) Representative multiplex immunofluorescence images demonstrating that AAV‐mediated SPHK1 knockdown reduces Schwann cell abundance in pancreatic tumors of KPC mice. Scale bars, 200 µm. Survival curves were analyzed using the log‐rank test. Data are presented as mean (SD) and were analyzed using Student's *t*‐test for two‐group comparisons or one‐way ANOVA followed by Tukey's post‐hoc test for multiple comparisons as appropriate. ^*^ *p* < 0.05, ^**^ *p* < 0.01, ^***^ *p* < 0.001.

To further prioritize functional candidates, we performed differential expression analysis comparing CAFs from PNI‐positive tumors versus PNI‐negative tumors. Among the 27 metabolic enzymes upregulated in stromal regions, we further identified seven metabolic enzymes upregulated in CAFs from PNI‐positive tumors (Figure 3A). Quantitative polymerase chain reaction (qPCR) confirmed the upregulation of all seven candidate metabolic enzymes in human primary CAFs from the PNI‐positive group compared with those from the PNI‐negative group (Figure 3B). Survival analysis identified four enzymes—SPHK1, SULF1, GFPT2, and PTGIS—as significantly associated with both overall survival and disease‐free survival (Figure S3A,B). Correlation analysis revealed strong associations between these enzymes and established PNI risk factors, supporting their pathogenic role in PNI (Figure S3C). To identify the primary driver enzyme of PNI, we overexpressed each candidate in HEK293T cells (Figure S3D) and functionally screened their conditioned media using a Transwell invasion assay with Schwann cells seeded in the lower chamber. Using this system as a gain‐of‐function screening platform, we evaluated the pro‐invasive potential of each candidate. Notably, SPHK1 emerged as the most potent inducer of cancer cell invasion, while other enzymes exhibited limited pro‐invasive activity (Figure 3C, Figure S3E). Gene Set Enrichment Analysis (GSEA) of TCGA‐PAAD tumors with high SPHK1 expression showed enrichment of “multicancer invasiveness signature”, “3ca metaprogram fibroblasts‐CAF”, “3ca metaprogram fibroblasts‐myofibroblasts”, and “3ca metaprogram fibroblasts lipid metabolism” pathways (Figure 3D, Figure S4A). In addition, Western blot analysis demonstrated that the expression of SPHK was markedly higher in three CAF isolates compared with normal human pancreatic duct epithelial (HPDE6c7) cells and pancreatic cancer cell lines SW1990 and AsPC‐1, supporting CAFs as a major source of SPHK1 in our study (Figure S4B). Importantly, our CAF subclustering analysis revealed that SPHK1 was predominantly expressed by the myCAF‐enriched cluster 0 in the human CRA001160 dataset (Figure S4C). We also integrated scRNA‐seq data from KPC mouse models to build a murine CAF atlas. Similarly, three CAF clusters were identified, with cluster 0 showing the highest myCAF signature scores, cluster 1 corresponding to iCAFs, and cluster 2 corresponding to apCAFs (Figure S4D,E). SPHK1 expression was again predominantly enriched in the myCAF‐enriched cluster 0 (Figure S4F).

To validate these findings in primary CAFs, we modulated SPHK1 expression in both human PDAC‐derived CAFs and KPC mouse‐derived CAFs (Figure S4G). Altering SPHK1 expression significantly changed the pro‐invasive effects of their conditioned medium on cancer cells (Figure 3E, Figure S4H). Because SPHK1 catalyzes the conversion of sphingosine to sphingosine‐1‐phosphate (S1P) in sphingolipid metabolism, we quantified extracellular S1P levels via enzyme‐linked immunosorbent assay (ELISA). ELISA confirmed elevated S1P secretion in conditioned media from SPHK1‐overexpressing CAFs across both human and murine systems (Figure 3F). As metabolites are conserved across species, we next examined whether murine KPC CAF‐derived SPHK1 metabolites could similarly promote invasion of human pancreatic cancer cells (Figure 3G, Figure S4I). The results showed that SPHK1 modulation in KPC CAFs also changed their conditioned medium's pro‐invasion effects on cancer cells. Notably, exogenous S1P supplementation rescued the impaired invasion caused by SPHK1 knockdown in CAFs, and direct treatment with exogenous S1P alone significantly enhanced cancer cell invasion (Figure 3H, Figure S4J). Although other metabolic enzymes may also contribute to the broader CAF secretome, these rescue experiments indicate that S1P is a major functional mediator of the SPHK1‐dependent pro‐invasive phenotype.

Immunofluorescence analysis confirmed SPHK1 expression in CAFs in both human and murine pancreatic tumor tissues (Figure 3I). Importantly, PNI‐positive tumors exhibited elevated SPHK1+αSMA+ cell abundance, and high abundance predicted worse overall survival in our tissue microarray cohort (Figure 3J,K). In vivo validation with the sciatic nerve invasion model demonstrated that modulation of SPHK1 in CAFs significantly influenced tumor burden, as assessed by bioluminescence imaging and gross examination (Figure 3L, Figure S4K). Functional evaluation further revealed a direct correlation between SPHK1 expression levels in CAFs and the severity of nerve dysfunction, quantified by sciatic nerve function index and limb function scores (Figure 3M,N). Histological analysis confirmed extensive neural encasement in SPHK1‐overexpressing CAF co‐injected mice compared with controls (Figure S4L).

To further evaluate the therapeutic relevance of SPHK1, we performed adeno‐associated virus (AAV)‐mediated knockdown of SPHK1 in the pancreas of KPC mice, thereby reducing SPHK1 expression within the pancreatic tumor microenvironment. Efficient knockdown at the tumor level was confirmed by Western blot analysis of whole‐tumor lysates (Figure S4M). Positron emission tomography/computed tomography (PET/CT) imaging showed a significant reduction in tumor burden after AAV‐mediated SPHK1 intervention (Figure 3O). Importantly, SPHK1 knockdown also led to decreased neural invasion within pancreatic tumors (Figure 3P, Figure S4N). These results indicate that inhibition of SPHK1 in the pancreatic tumor microenvironment can restrain PDAC progression and perineural invasion in vivo, supporting SPHK1 as a therapeutically relevant target for mitigating PNI in PDAC.

### 2.4 Sphingosine‐1‐Phosphate Transcriptionally Upregulates MALL to Trigger Amoeboid Transition

**FIGURE 4:**
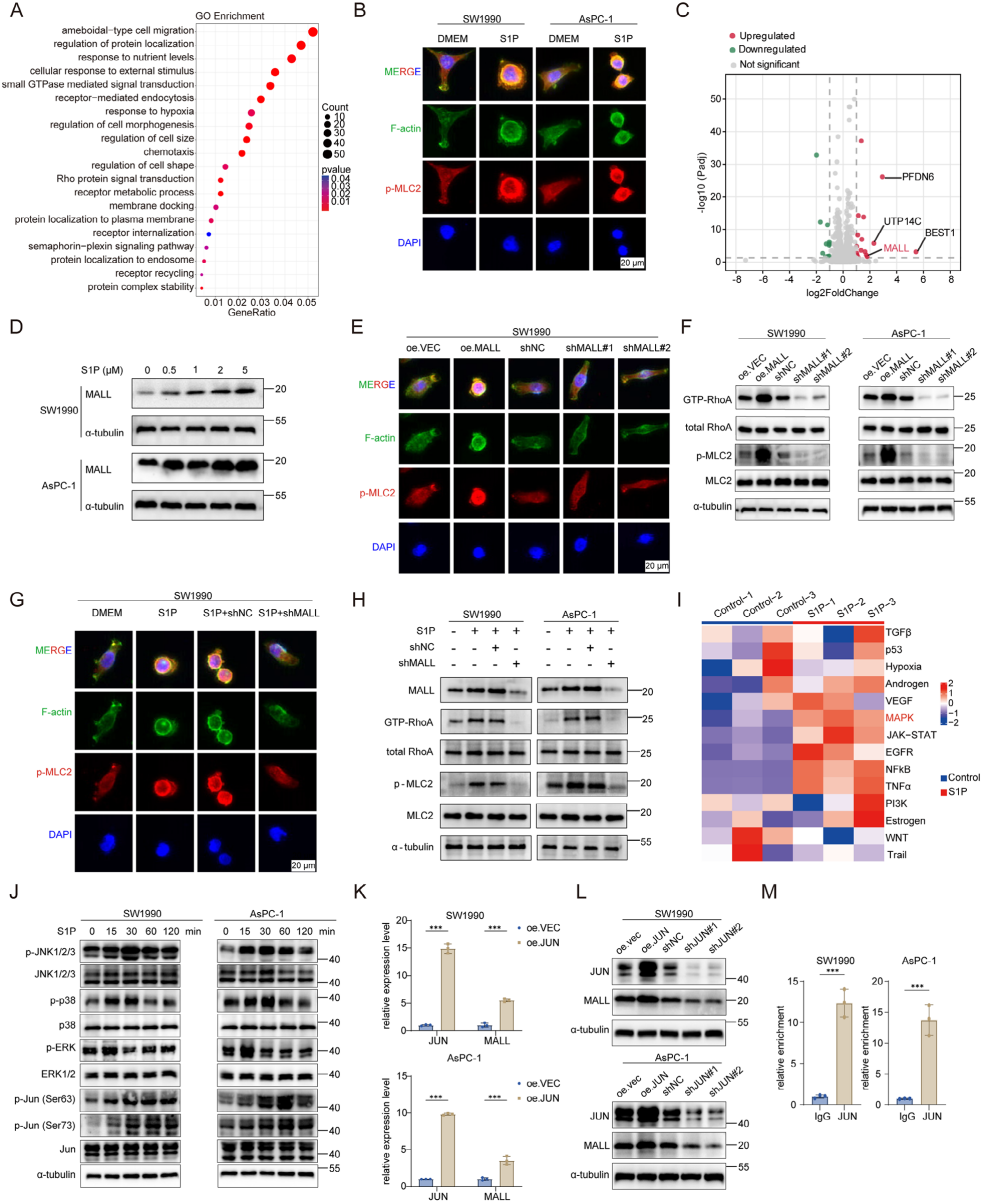
Sphingosine‐1‐phosphate transcriptionally upregulates MALL to trigger amoeboid transition. A) Gene Ontology functional enrichment analysis of S1P‐treated SW1990 cells (5 µm). B) Immunofluorescence staining for phosphorylated MLC2 and F‐actin demonstrating amoeboid transition upon S1P treatment (5 µm) compared with vehicle control. Scale bars, 20 µm. C) Volcano plot showing that MALL is significantly upregulated following S1P treatment (5 µm). D) Western blot validation demonstrating that MALL expression is significantly upregulated by S1P treatment in a dose‐dependent manner. E) Functional validation showing that MALL gain‐of‐function promotes amoeboid transition. Scale bars, 20 µm. F) Western blot analysis demonstrating that MALL gain‐of‐function activates the phosphorylated MLC2 signaling pathway. G, H) S1P‐induced amoeboid transition (5 µm) occurs in a MALL‐dependent manner as demonstrated by immunofluorescence (G) and Western blot (H). Scale bars, 20 µm. I) PROGENy pathway activity profiling showing activation of the MAPK signaling pathway in SW1990 cells following S1P treatment (5 µm). J) Time‐course Western blot analysis showing that S1P treatment (5 µm) rapidly activates JNK and the downstream transcription factor JUN, with modest effects on the ERK and p38 MAPK pathways. K) JUN overexpression alters MALL expression as determined by qPCR. L) JUN overexpression and JUN knockdown alter MALL expression as shown by Western blot. M) Chromatin immunoprecipitation followed by qPCR confirming direct JUN binding to the endogenous MALL promoter region in S1P‐treated cells. Data are presented as mean (SD) and were analyzed using Student's t‐test for two‐group comparisons or one‐way ANOVA followed by Tukey's post‐hoc test for multiple comparisons as appropriate. ^*^ *p* < 0.05, ^**^ *p* < 0.01, ^***^ *p* < 0.001.

To understand how S1P promotes PNI, we sought to identify the downstream molecular mechanisms. GO enrichment analysis of S1P‐treated SW1990 cells revealed significant activation of migration‐related pathways, with particular enrichment in amoeboid movement and cell motility‐related pathways (Figure 4A). These pathway enrichment findings prompted our investigation of whether S1P directly induces amoeboid transition in cancer cells. Immunofluorescence staining demonstrated that S1P treatment induced amoeboid morphological transition, characterized by rounded cell shape, enrichment of F‐actin at the cell periphery, and markedly elevated phosphorylated myosin light chain 2 (p‐MLC2)—hallmarks of amoeboid movement (Figure 4B). This observation supports recent findings that cancer cells utilize amoeboid migration during perineural invasion to facilitate invasion through dense stromal environments [18].

To identify the key targets that mediate S1P‐induced amoeboid transition, differential expression analysis was performed to identify significantly upregulated candidates following S1P treatment (Figure 4C). To prioritize candidates for further investigation, we evaluated the top four upregulated genes (MALL, PFDN6, BEST1, and UTP14C) according to tumor‐versus‐normal tissue expression, survival association, and relevance to the PNI phenotype. Among these candidates, MALL showed the strongest convergence of evidence: it was increased in PDAC tissues (Figure S5A), associated with worse overall and disease‐free survival (Figure S5B,C), and specifically upregulated in cancer cells from PNI‐positive tumors in the CRA001160 dataset (Figure S5D). From a biological perspective, MAL family proteins are known to regulate vesicular trafficking of transmembrane proteins and maintain dynamic homeostasis of cell surface proteins. These functions align intrinsically with the high membrane plasticity required for amoeboid morphological transition. Consequently, MALL was prioritized for further investigation. qPCR and Western blot analyses demonstrated that MALL expression was upregulated by S1P treatment in a dose‐dependent manner (Figure 4D, Figure S6A). Functional studies showed that MALL overexpression promoted amoeboid morphological transition and activated the RhoA/p‐MLC2 pathway (Figure 4E,F; Figure S6B), whereas MALL depletion abrogated S1P‐induced amoeboid transition (Figure 4G,H, Figure S6C). GSEA analysis showed enrichment of migration and motility pathways in MALL‐high tumors (Figure S6D). Together, these findings identify MALL as a clinically and functionally relevant downstream effector of S1P‐driven amoeboid plasticity.

To elucidate the signaling pathway connecting S1P stimulation to MALL transcriptional upregulation, we first applied PROGENy pathway analysis, which highlighted MAPK signaling as the most consistently activated pathway across biological replicates (Figure 4I). Time‐course experiments showed rapid and sustained JNK and JUN phosphorylation following S1P treatment, whereas ERK and p38 exhibited modest and transient activation (Figure 4J). Pharmacological inhibition with selective MAPK inhibitors revealed that JNK blockade specifically abrogated S1P‐induced MALL upregulation, while inhibition of ERK or p38 had minimal effects, establishing the JNK pathway as the critical mediator (Figure S6E). While PROGENy profiling also revealed modest activation of JAK/STAT, EGFR, and TNFα/NF‐κB‐related inflammatory programs, pharmacological inhibition of these pathways failed to markedly attenuate S1P‐induced MALL upregulation (Figure S6F), with NF‐κB blockade serving as a representative validation for the highly concordant inflammatory TNFα/NF‐κB activation. Expression profiling of S1P receptors identified sphingosine‐1‐phosphate receptor 3 (S1PR3) as the predominant receptor subtype in pancreatic cancer cells (Figure S6G). Functional validation using S1PR3 knockdown and pharmacological inhibition demonstrated that S1PR3 depletion significantly diminished S1P‐induced JNK activation and MALL upregulation, establishing S1PR3 as the primary receptor mediating S1P effects on cancer cell motility (Figure S6H).

Intervention experiments revealed that JUN modulation directly altered MALL expression levels, with JUN overexpression elevating and JUN knockdown reducing MALL expression levels by quantitative PCR and Western blot (Figure 4K,L, Figure S6I). This regulatory relationship was further supported by a significant positive correlation between JUN and MALL expression in the TCGA‐PAAD cohort (Figure S6J). To confirm direct transcriptional regulation, chromatin immunoprecipitation assays confirmed direct JUN binding to the endogenous MALL promoter region (Figure 4M). These findings demonstrate that CAF‐derived S1P promotes the amoeboid migration phenotype essential for perineural invasion via S1PR3/JNK/JUN‐mediated MALL upregulation.

### 2.5 MALL Binds to Syndecan‐4 and Promotes Amoeboid Transition

**FIGURE 5:**
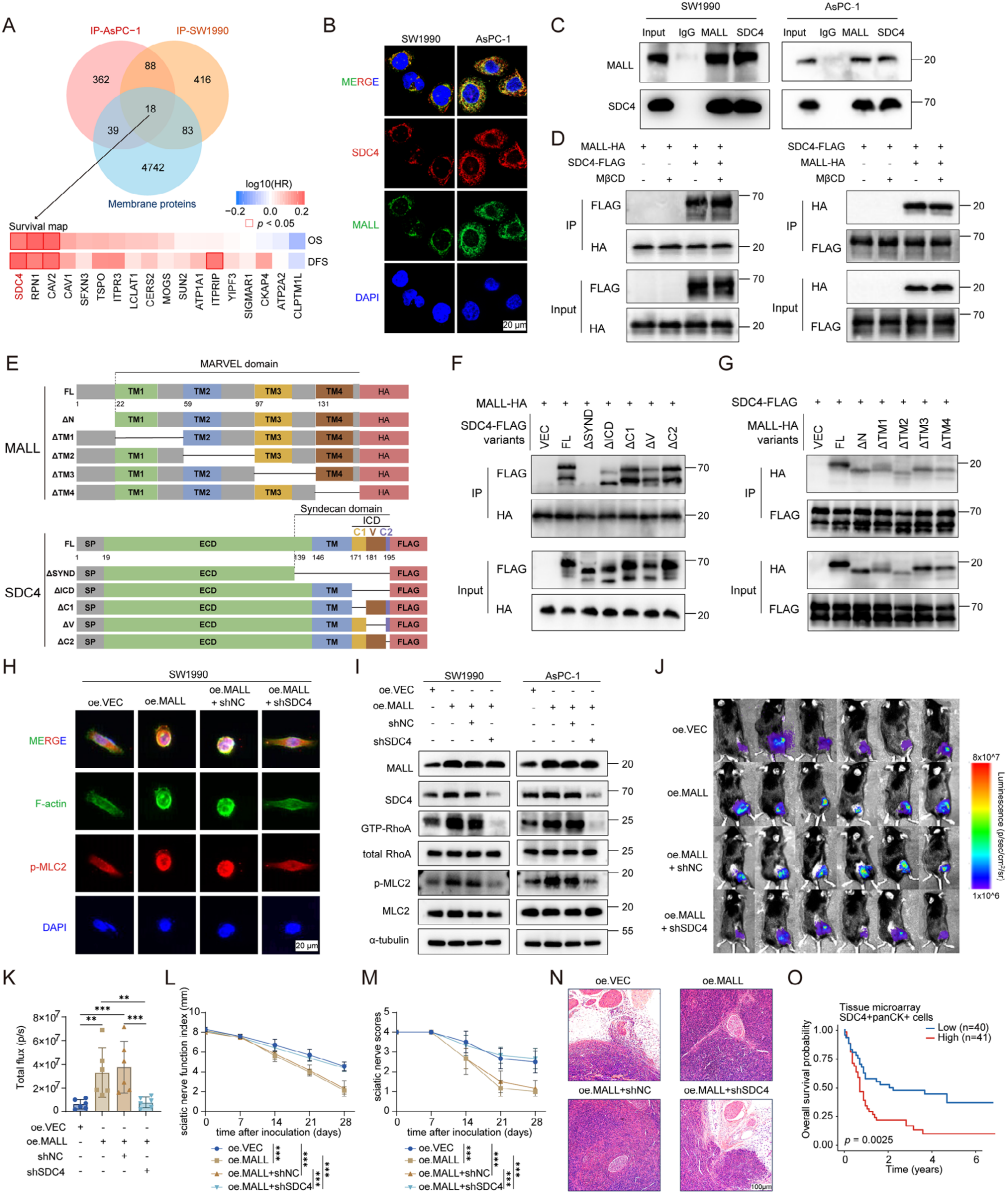
MALL binds to syndecan‐4 and promotes amoeboid transition. A) Venn plot showing the overlapping MALL‐interacting membrane proteins identified by immunoprecipitation‐mass spectrometry, and their association with OS and DFS in the TCGA dataset. In the lower survival map, statistically significant associations (*p* < 0.05) are highlighted by red boxes. B) Confocal immunofluorescence microscopy demonstrating co‐localization of MALL and SDC4 proteins in cancer cells. Scale bars, 20 µm. C) Reciprocal co‐immunoprecipitation validating the interaction between endogenous MALL and SDC4 proteins. D) Lipid raft disruption assay using methyl‐β‐cyclodextrin (MβCD, 10 µm) followed by reciprocal co‐immunoprecipitation. E) Schematic diagrams illustrating the domain structures of MALL and SDC4 proteins, highlighting functional domains and predicted interaction regions. F) Co‐immunoprecipitation analysis using full‐length MALL with various SDC4 truncation mutants to map the MALL‐binding domain within SDC4. G) Co‐immunoprecipitation analysis using full‐length SDC4 with various MALL truncation mutants to identify the SDC4‐binding domain within MALL. H, I) Sequential perturbation experiments combining MALL overexpression with SDC4 knockdown demonstrating that SDC4 loss suppresses MALL‐induced amoeboid transition (H) and phosphorylated MLC2 pathway activation (I). Scale bars, 20 µm. J‐M) In vivo sciatic nerve invasion assay under sequential perturbation conditions showing that SDC4 loss significantly blunts MALL‐driven neural invasion as measured by bioluminescence signal intensity (J, K), sciatic nerve function index (L), and limb scores (M). N) Representative hematoxylin and eosin staining images of tumors from the sciatic nerve invasion model demonstrating differential invasion patterns. Scale bars, 100 µm. O) Kaplan‐Meier survival curves for overall survival stratified by SDC4+panCK+ cell abundance in the PDAC tissue microarray cohort (n = 81). panCK, pan‐cytokeratin. Survival curves were analyzed using the log‐rank test. Data are presented as mean (SD) and were analyzed using Student's *t*‐test for two‐group comparisons or one‐way ANOVA followed by Tukey's post‐hoc test for multiple comparisons as appropriate. ^*^ *p* < 0.05, ^**^ *p* < 0.01, ^***^ *p* < 0.001.

Although MALL emerged as a key mediator of S1P‐induced amoeboid transition, the mechanism by which it promotes this phenotype remained unclear. MALL is a member of the MAL gene family, which regulates membrane protein transport and signal transduction pathways [21]. Similar to other family members (MAL and MAL2), MALL shuttles with its interacting proteins in vesicular structures between the cell membrane, endosomes, and Golgi apparatus, participating in maintaining the homeostasis of cell surface proteins [22]. Based on this functional characteristic, we hypothesized that MALL exerts its biological effects through protein‐protein interactions and sought to identify its binding partners. To test this hypothesis, we performed immunoprecipitation followed by mass spectrometry (IP‐MS) analysis on pancreatic cancer cells stably overexpressing MALL‐HA with particular focus on membrane proteins. This approach identified 18 proteins as potential MALL‐interacting membrane proteins. Among these candidates, SDC4, RPN1, and CAV2 were significantly associated with poor OS and DFS in the TCGA dataset, as visualized in the prognostic survival map (Figure 5A, Figure S7A). Notably, SDC4, an upstream regulator of canonical RhoA signaling that participates in amoeboid movement and contributes to cell migration and invasion processes, emerged as a prominent binding partner given its role in cancer metastasis (Figure 5A, Figure S7A) [23, 24].

Confocal microscopy validated the co‐localization of MALL and SDC4, and reciprocal co‐immunoprecipitation further demonstrated a robust cellular association between the two proteins (Figure 5B,C). Previous studies have shown that MAL family proteins can associate with lipid rafts to facilitate vesicular trafficking. To test whether the association between MALL and SDC4 depends on lipid rafts, we performed lipid raft disruption assays using methyl‐β‐cyclodextrin (MβCD) before co‐immunoprecipitation. MβCD treatment did not substantially diminish the MALL–SDC4 association, suggesting that the observed association cannot be solely explained by lipid raft integrity (Figure 5D). In silico molecular docking analysis also suggested a plausible interaction interface between MALL and SDC4, with a docking score of –388.94 and a confidence score of 0.9917, which is empirically classified by HDOCK as “very likely to bind” (Figure S7B; the predicted interface residues and hydrogen‐bonding contacts are summarized in Table S2). To investigate the crucial interaction domains between MALL and SDC4, we constructed domain deletion mutant plasmids based on their wild‐type protein domain structures and performed reciprocal co‐immunoprecipitation experiments (Figure 5E). Results showed that deletion of the Syndecan domain in SDC4 markedly disrupted their interaction (Figure 5F). However, deletion mutations of individual sub‐domains within the Syndecan domain did not show significant interaction disruption; only weakened interactions were observed with ICD or V subdomain deletions, while C1 or C2 subdomain deletions did not seem to affect the interaction. To further refine the MALL‐binding region within SDC4, we generated additional deletion mutants lacking either the transmembrane domain (ΔTM) or the amino acid 139–170 region (ΔAA139–170), which encompasses the transmembrane segment. Both mutants showed a marked loss of binding to MALL, supporting the conclusion that the transmembrane‐containing 139–170 region of SDC4 is critical for this association (Figure S7C). To further examine the MALL‐associated region involved in SDC4 binding, we generated a series of MALL deletion variants and performed co‐immunoprecipitation assays. Although the different MALL mutants showed varying degrees of reduced association with SDC4, no single deletion was sufficient to abolish co‐immunoprecipitation under our experimental conditions (Figure 5G). These results, therefore, do not define a discrete MALL binding site. Instead, they suggest that the multi‐pass membrane architecture or overall structural integrity of MALL may contribute to its efficient association with SDC4.

To determine whether SDC4 was functionally required for MALL‐mediated effects, we conducted sequential perturbation experiments. SDC4 knockdown suppressed MALL‐induced amoeboid morphology and p‐MLC2 activation (Figure 5H,I, Figure S7D–G). Conversely, overexpression of SDC4 in MALL‐knockdown cells restored amoeboid transition and RhoA/p‐MLC2 signaling in vitro (Figure S7H,I). Given that SDC4 is an established upstream regulator of RhoA signaling, we next examined whether the MALL‐induced amoeboid transition functionally depended on downstream RhoA pathway activity [24]. Inhibition of the downstream effector of the RhoA pathway with Y‐27632 markedly attenuated the increase in p‐MLC2 and cytoskeletal remodeling induced by MALL overexpression (Figure S8A,B), functionally linking the MALL–SDC4 module to the RhoA/p‐MLC2 amoeboid program. In vivo, SDC4 knockdown significantly blunted MALL‐driven neural invasion in the sciatic nerve invasion model, as measured by bioluminescence, gross examination, sciatic nerve function index, limb scores, and histology (Figure 5J–N, Figure S7J). High SDC4+panCK+ cell abundance in human tumors also predicted worse OS (Figure 5O). These findings establish SDC4 as a key downstream effector of MALL during neural invasion.

### 2.6 MALL Promotes SDC4 Endosomal Recycling and Stabilization

**FIGURE 6:**
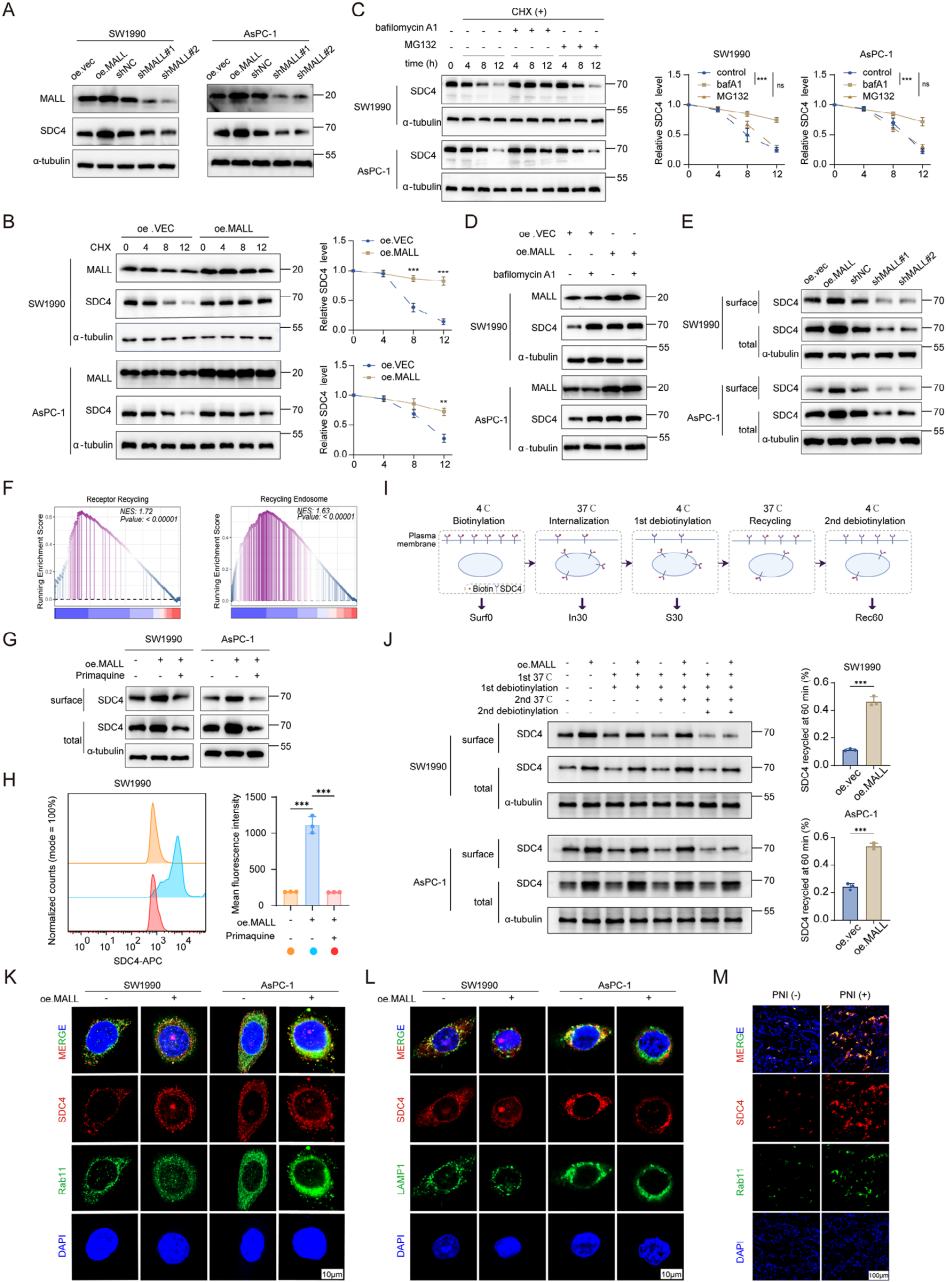
MALL promotes SDC4 endosomal recycling and stabilization. A) Western blot analysis demonstrating the effect of MALL modulation on total SDC4 protein levels in cancer cells. B) Cycloheximide chase assay (Cycloheximide, CHX; 100 µg/mL) in control and MALL‐overexpressing cancer cells, demonstrating that MALL overexpression significantly elevates SDC4 protein stability. C) Cycloheximide chase assay performed with or without the proteasome inhibitor MG132 (10 µm) or the lysosome inhibitor Bafilomycin A1 (200 nM). D) The effect of Bafilomycin A1 treatment (200 nM) and/or MALL overexpression on SDC4 protein levels. E) The effect of MALL modulation on total and surface SDC4 protein levels. F) GSEA analysis of tumors with high MALL expression in the TCGA dataset. G, H) Cell surface biotinylation assay (50 µm, 12 h) (G) and flow cytometry (300 µm, 1 h) (H) showing that treatment with the recycling inhibitor primaquine blocks MALL‐mediated increase in SDC4 surface expression. I) Schematic diagram of the cell surface biotinylation assay used to measure protein recycling. J) Cell surface biotinylation assay comparing control and MALL‐overexpressing cancer cells, showing that MALL overexpression significantly increases the SDC4 recycling rate. K, L) Confocal immunofluorescence microscopy showing that MALL overexpression enhances SDC4 co‐localization with recycling endosome markers (K) while reducing co‐localization with lysosomal markers (L). Scale bars, 10 µm. M) Representative tissue immunofluorescence images demonstrating co‐localization of SDC4 with the recycling endosome marker Rab11 in human PDAC specimens. Scale bars, 100 µm. Data are presented as mean (SD) and were analyzed using Student's *t*‐test for two‐group comparisons or one‐way ANOVA followed by Tukey's post‐hoc test for multiple comparisons as appropriate. ^*^ *p* < 0.05, ^**^ *p* < 0.01, ^***^ *p* < 0.001.

To complete our mechanistic understanding, we investigated how MALL regulates SDC4 function at the molecular level. Initial analysis showed that MALL modulation affected total SDC4 protein levels (Figure 6A). Cycloheximide chase assays demonstrated that MALL overexpression prolonged SDC4 protein stability (Figure 6B). This result indicated that MALL protects SDC4 from degradation.

To investigate the mechanism underlying SDC4 stabilization, we treated cells with proteasome or lysosome inhibitors to delineate the relevant degradation pathways. Cycloheximide chase assays conducted with or without the proteasome inhibitor MG132 or the lysosome inhibitor bafilomycin A1 demonstrated that MALL selectively impedes lysosomal degradation and thereby stabilizes SDC4 protein levels (Figure 6C). MALL overexpression elevated SDC4 protein to levels comparable to those observed with Bafilomycin A1 treatment alone, and the combination of MALL overexpression and Bafilomycin A1 treatment yielded no additional increase in SDC4 protein levels beyond either treatment alone, indicating that MALL and lysosomal inhibition operate through the same pathway (Figure 6D). Notably, surface biotinylation assays revealed that MALL modulation exerted a more pronounced effect on cell surface SDC4 levels, suggesting that MALL‐mediated SDC4 stabilization is particularly critical for maintaining SDC4 at the plasma membrane (Figure 6E).

GSEA analysis showed that tumors with high MALL expression were enriched for receptor recycling and recycling endosomes pathways (Figure 6F). This finding is consistent with the established role of MAL family proteins in membrane trafficking and receptor recycling processes [21]. Surface biotinylation assays and flow cytometry validated that treatment with the recycling inhibitor primaquine abrogated MALL‐driven SDC4 surface accumulation (Figure 6G,H, Figure S9A). We then used surface biotinylation assays to assess the endocytosis and recycling of plasma membrane SDC4 (Figure 6I). Our results revealed that MALL overexpression did not affect the SDC4 internalization rate compared with control cells; however, it significantly enhanced the SDC4 recycling rate (increasing from 33% to 54% in SW1990 cells and from 20% to 48% in AsPC‐1 cells) (Figure 6J, Figure S9B). These findings demonstrate that MALL promotes the return of SDC4 to the plasma membrane rather than its initial endocytic uptake. Confocal microscopy revealed that MALL overexpression enhanced SDC4 co‐localization with Rab11‐positive recycling endosomes and reduced its presence in lysosomal compartments (Figure 6K,L), providing mechanistic insight into SDC4 trafficking. Clinical validation in human specimens further demonstrated SDC4‐Rab11 co‐localization, underscoring the pathophysiological relevance of this cycling mechanism (Figure 6M, Figure S9C). These results suggested that MALL promotes SDC4 recycling away from degradative pathways to stabilize SDC4.

### 2.7 SDC4‐PTN‐Mediated Cancer Cell–Schwann Cell Crosstalk Amplifies Perineural Invasion

**FIGURE 7:**
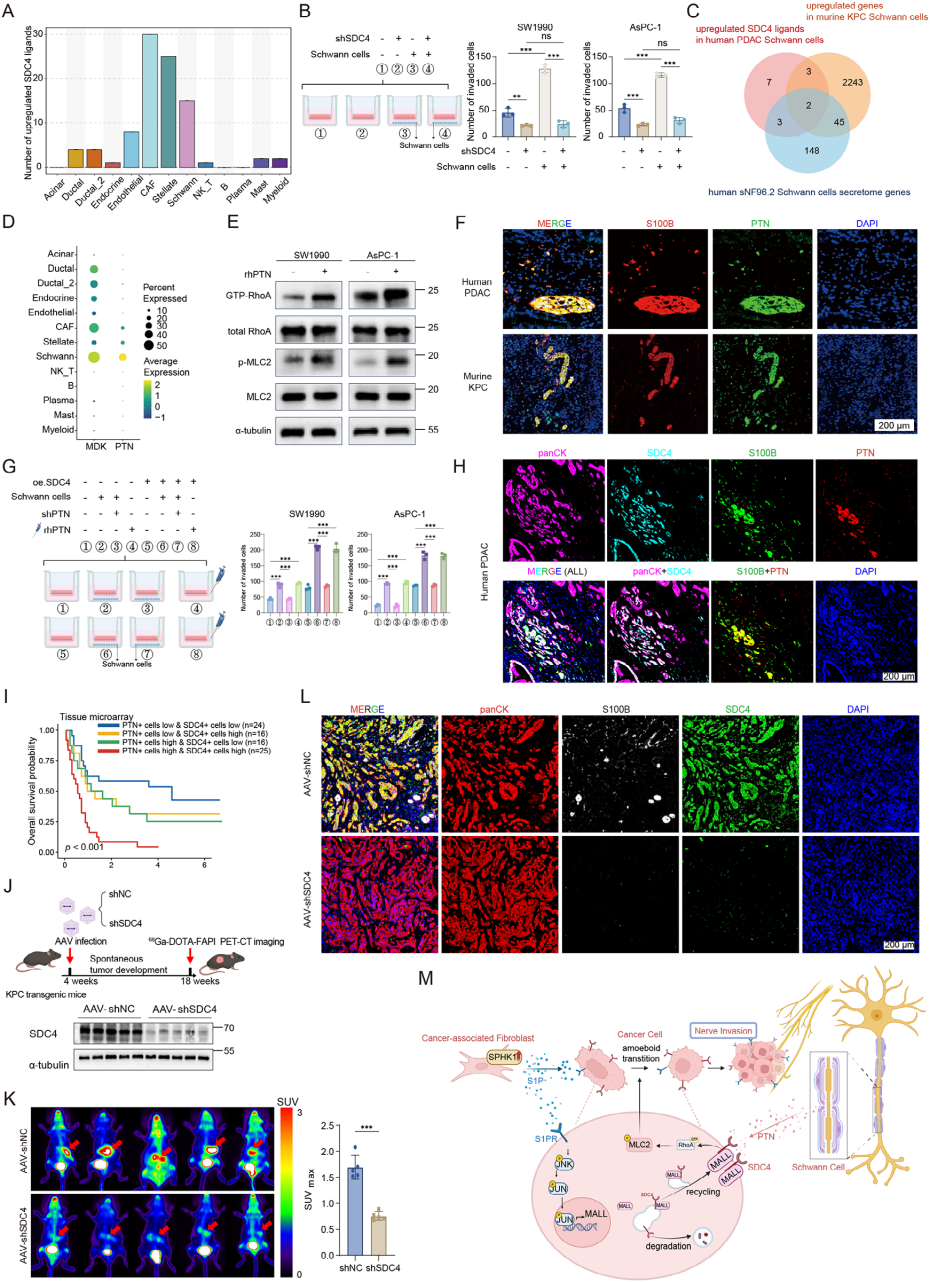
SDC4–PTN mediated cancer cell–Schwann cell crosstalk amplifies perineural invasion. A) Bar plot showing the number of upregulated SDC4 ligands across different cell types. B) Transwell invasion assays performed with or without Schwann cells in combination with cancer cell SDC4 knockdown. C) Venn diagram demonstrating the overlapping SDC4 ligands. D) Dot plot indicating cell type specificity and expression levels of the SDC4 ligands MDK and PTN. E) Western blot analysis showing GTP‐bound RhoA and p‐MLC2 levels following PTN stimulation (100 ng/ml). F) Representative immunofluorescence images demonstrating that PTN is predominantly expressed by Schwann cells in human and mouse pancreatic cancer tissue sections. Scale bars, 200 µm. G) Functional dissection of SDC4–PTN axis in mediating cancer cell–Schwann cell interactions using Transwell co‐culture invasion models. PTN: 100 ng/ml. H) Multiplex immunofluorescence showing the spatial distribution patterns of SDC4 and PTN expression in tumor and nerve regions, demonstrating their co‐localization at sites of neural invasion. Scale bars, 200 µm. I) Tissue microarray survival analysis demonstrating that patients with dual‐high PTN and SDC4 expression exhibited the worst OS compared with other patients. J) Western blot analysis confirming adeno‐associated virus‐mediated pancreatic SDC4 knockdown in KPC mice. K) PET/CT imaging analysis demonstrating significantly reduced tumor burden following SDC4‐targeted intervention in KPC mice. L) Multiplex immunofluorescence analysis showing decreased neural invasion in pancreatic tumors after SDC4‐targeted intervention. Scale bars, 200 µm. M) Schematic diagram illustrating the CAF‐derived S1P–S1PR3/JNK/JUN–MALL–SDC4 signaling axis. Survival curves were analyzed using the log‐rank test. Data are presented as mean (SD) and were analyzed using Student's t‐test for two‐group comparisons or one‐way ANOVA followed by Tukey's post‐hoc test for multiple comparisons as appropriate. ^*^ *p* < 0.05, ^**^ *p* < 0.01, ^***^ *p* < 0.001.

Although SDC4 promoted cancer cell amoeboid movement and neural invasion as an upstream regulator of RhoA signaling, the mechanism by which SDC4 facilitates neural invasion remains to be further elucidated. As a multi‐ligand cell‐surface proteoglycan, SDC4 can bind to diverse cytokines, chemokines, and other microenvironmental ligands, coordinate cellular responses to surrounding signals, and regulate cellular functions, including cytoskeletal reorganization [25]. This suggests that SDC4 may play a key mediating role in enhancing cancer cell‐microenvironment interactions to promote pancreatic cancer neural invasion. We compiled known SDC4 ligands from multiple ligand‐receptor databases (Table S3) and analyzed the number of upregulated SDC4 ligands across different cell types in single‐cell datasets. The results showed that Schwann cells represent a major source of SDC4 ligands (Figure 7A, Figure S10A). Consistent with this observation, the ability of Schwann cells to enhance cancer cell invasion in Transwell assays was significantly attenuated by SDC4 knockdown in cancer cells, suggesting that SDC4 promotes perineural invasion in part by facilitating cancer cell–Schwann cell communication (Figure 7B, Figure S10B).

We hypothesized that SDC4 might facilitate cancer cell–Schwann cell interactions by binding to Schwann cell‐derived ligands. To identify the relevant Schwann cell‐derived ligands, we intersected upregulated SDC4 ligands in human PDAC Schwann cells, secreted proteins from sNF96.2 Schwann cell culture medium, and upregulated genes in mouse KPC Schwann cells. This yielded two candidate ligands: pleiotrophin (PTN) and midkine (MDK) (Figure 7C). Analysis of multiple bulk RNA sequencing datasets confirmed their expression in Schwann cells (Figure S10C). Single‐cell data analysis showed that PTN exhibited higher and more Schwann cell‐specific expression, whereas MDK showed a broader distribution across multiple cell types (Figure 7D). Moreover, PTN treatment alone increased GTP‐bound RhoA and p‐MLC2 levels and induced cytoskeletal remodeling consistent with amoeboid transition, indicating that PTN is sufficient to activate the downstream motility program in pancreatic cancer cells (Figure 7E, Figure S10D). Immunofluorescence staining confirmed PTN expression by Schwann cells in both human and mouse pancreatic cancer tissues (Figure 7F, Figure S10E). This highly Schwann cell‐specific expression pattern made PTN particularly attractive as it would mediate more specific cancer cell–Schwann cell interactions compared with ligands broadly expressed by multiple cell types. Combined with prior evidence linking PTN to pancreatic cancer progression and poor clinical outcome—including its elevated expression in 63.2% of pancreatic cancer specimens and its clinical correlation with perineural invasion and reduced survival [26]‐these convergent data position PTN as a key candidate for mediating SDC4‐dependent interactions between cancer cells and Schwann cells.

We next tested whether the SDC4–PTN axis functionally mediates this interaction. PTN knockdown in Schwann cells significantly reduced their ability to promote cancer cell invasion, whereas supplementation with recombinant PTN restored this pro‐invasive activity (Figure 7G, Figure S10F). These findings support a model in which S1P‐upregulated SDC4 not only enhances baseline amoeboid motility but also increases cancer cell responsiveness to Schwann cell‐derived PTN, thereby amplifying this pro‐invasive effect by coordinating cell‐intrinsic motility enhancement with microenvironmental interactions that drive perineural invasion.

Multiplex immunofluorescence provided additional support for the SDC4–PTN axis by showing spatial proximity of SDC4 and PTN at sites of neural invasion (Figure 7H, Figure S10G). This spatial relationship supports the functional interaction between SDC4‐expressing cancer cells and PTN‐expressing Schwann cells in PNI‐positive tumors. Clinically, patients with dual‐high PTN and SDC4 expression showed the poorest OS in the tissue microarray cohort (Figure 7I), and this relationship was recapitulated for both OS and DFS in TCGA‐PAAD (Figure S10H,I). Finally, in vivo AAV‐mediated SDC4 knockdown in the pancreas of KPC mice reduced tumor burden and neural invasion (Figure 7J–L, Figure S10J), validating SDC4 as a key driver of PNI and demonstrating its therapeutic potential as a targetable mediator of perineural invasion in PDAC.

Collectively, these findings support a multi‐cellular signaling cascade in which CAF‐derived S1P activates S1PR3/JNK/JUN signaling to upregulate MALL in cancer cells. MALL increases surface SDC4 by promoting its recycling and limiting lysosomal degradation, thereby enhancing RhoA/p‐MLC2‐dependent amoeboid motility and cancer cell–Schwann cell communication to promote perineural invasion (Figure 7M). This model elucidates how metabolic crosstalk in the tumor microenvironment drives the aggressive neural invasion characteristic of pancreatic cancer.

## 3 Discussion

PDAC is characterized by aggressive local spread, and perineural invasion is one of its most clinically relevant pathological hallmarks, because it contributes to severe pain, local recurrence, and poor survival [14]. Yet the mechanisms that make the pancreatic tumor microenvironment so permissive for neural invasion remain incompletely understood. Here, we identify a metabolite‐driven signaling axis that functionally connects CAF activity to Schwann cell‐supported neural invasion, and thereby provides a mechanistic framework for how stromal metabolism is coupled to neurotropism in PDAC.

Our study confirmed a significant cooperation of CAFs and Schwann cells in PNI‐positive PDAC tumors by integrating single‐cell, bulk transcriptomics, and clinical analyses. The abundance of these two stromal cell types showed a positive correlation, and their combined presence predicted poorer patient outcomes. The identification of CAFs as a primary stromal driver in PNI represents a significant advance, aligning with recent studies suggesting that stromal cells actively cooperate to promote PNI rather than functioning as passive bystanders [15, 16]. Our single‐cell subclustering analysis further revealed that this pro‐invasive stromal response is not uniform and is characterized by the expansion of a myCAF‐enriched subpopulation. Moreover, we demonstrate that CAFs in PNI‐positive tumors upregulate multiple metabolic enzymes, indicating their role as a major source of tumor‐promoting metabolites within the perineural niche and supporting the concept of localized stromal adaptation for neural invasion [15]. Beyond the established role of CAFs in providing metabolic fuel like glutamine or lipids, we demonstrate that CAFs secrete signaling metabolite S1P, generated by SPHK1, to directly stimulate cancer cell invasion [9, 27]. While the SPHK1/S1P axis has been studied in other cancer contexts, its upregulation in CAFs and functional role in PDAC PNI had not been previously established [28, 29]. Given that AAV‐mediated SPHK1 knockdown reduced both tumor burden and neural invasion in KPC mice, this pathway appears to represent a tractable upstream vulnerability. An important direction for future work is to identify the upstream signals responsible for SPHK1 induction in PNI‐associated CAFs. Because our additional subtype analysis showed that SPHK1 is predominantly enriched in the myCAF population, myCAF‐associated cues may contribute to its upregulation. In this context, TGF‐β represents one plausible upstream trigger, as prior studies have shown that TGF‐β can induce SPHK1 in fibroblasts and promote myofibroblast‐like differentiation [30, 31].

We found that CAF‐derived S1P triggers a transition to amoeboid migration in cancer cells. This form of movement depends on RhoA–ROCK–myosin II–driven contractility rather than proteolytic activity, facilitating rapid progression through constrained environments such as the dense PDAC stroma [17, 18, 23, 32]. Recent studies have directly connected amoeboid phenotype with rapid nerve invasion, and our work identifies the CAF–S1P axis as an upstream metabolic trigger of this amoeboid switch [18]. To elucidate the downstream signaling events, we established that S1P acts through the S1PR3–JNK–JUN pathway to drive transcriptional upregulation of MALL. While MAPK signaling is widely recognized in PDAC progression, the specific pathways active in PNI remain poorly defined. In PDAC, ERK typically mediates KRAS‐driven proliferation, whereas JNK activation is linked to stress‐induced invasion programs [33, 34, 35, 36, 37]. Our study delineates a signaling cascade from extracellular S1P to nuclear JUN activation and subsequent MALL expression, revealing a JNK‐dependent pathway that coordinates this invasive behavior and establishes a previously unrecognized role for MALL in promoting invasion and metastasis. MAL family proteins are known to regulate endosomal trafficking and membrane protein sorting, thereby influencing cell signaling through maintaining membrane protein homeostasis [21, 22]. Our discovery that MALL stabilizes SDC4 by redirecting it from lysosomal degradation toward Rab11‐positive recycling endosomes aligns with this paradigm. Although MAL family proteins are closely linked to lipid raft‐associated trafficking, our lipid raft disruption experiments suggest that the MALL–SDC4 association observed here cannot be explained solely by localization within lipid raft microdomains. Elevated surface SDC4 sustains RhoA/p‐MLC2 signaling to reinforce amoeboid motility, and it heightens responsiveness to microenvironmental cues in the PNI niche. SDC4, as a core proteoglycan receptor, binds multiple Schwann cell‐secreted ligands, including TGF‐β, MDK, and PTN [25, 38, 39, 40]. Among these, PTN is specifically and highly expressed by Schwann cells, positioning it as a selective mediator of this intercellular interaction. Building on prior studies associating elevated PTN expression in PDAC with PNI and poor prognosis, our results demonstrate that the SDC4–PTN axis serves as a key mediator of cancer cell–Schwann cell communication [26]. Schwann cell–derived PTN binding to cancer cell–surface SDC4 promotes directional cancer cell invasion toward nerves, completing a coordinated biological pathway from CAF‐initiated signaling to Schwann cell–guided neural invasion. Our work thereby connects the intrinsic invasive machinery of cancer cells with microenvironmental interactions that direct neural invasion.

Several limitations merit consideration. First, while our in vitro and murine models provide robust mechanistic insights, they may not fully recapitulate the complexity of the human PDAC microenvironment. Second, although we identified Schwann cells as the critical source of PTN that engages cancer cell‐expressed SDC4, we did not comprehensively characterize Schwann cell heterogeneity. Emerging evidence suggests Schwann cells can adopt distinct functional states, such as repair‐like or myelinating phenotypes, which may differentially influence nerve invasion. In our current model, Schwann cells function primarily as a microenvironmental source of ligands (PTN) and a structural scaffold for invasion. Given that our bulk and single‐cell analyses confirmed robust PTN expression across Schwann cell populations and established its functional necessity in the SDC4 axis, the specific contributions of distinct Schwann cell subsets were not the primary focus of this metabolic signaling investigation. Because Schwann cells are embedded within the dense desmoplastic stroma and peripheral nerves, they are difficult to dissociate efficiently and are often captured at low abundance in conventional single‐cell datasets. Future studies combining single‐nucleus RNA sequencing, spatial transcriptomics, and Schwann cell‐enriched, or Schwann cell‐sorted, profiling should help resolve distinct Schwann cell states within the perineural niche and define how these states influence ligand production and cancer‐nerve interactions. Finally, regarding our in vivo therapeutic intervention, we utilized AAV‐mediated knockdown to reduce SPHK1 expression in the pancreas of KPC mice. We acknowledge that this approach may reduce SPHK1 levels in non‐CAF compartments. However, our co‐injection experiments—where genetic perturbation was restricted exclusively to CAFs—recapitulated the phenotypes observed in the AAV‐treated KPC mice, thereby reinforcing a CAF‐dependent mechanism. Furthermore, the AAV‐mediated knockdown mimics the systemic, non‐cell‐type‐specific effects of potential pharmacological inhibitors, providing a valid “proof‐of‐concept” for targeting the SPHK1 axis therapeutically.

In summary, this study delineates a multi‐step, multi‐cellular signaling cascade driving PDAC PNI: from CAF‐derived S1P to the activation of the cancer cell invasive machinery, culminating in amplified cancer–Schwann cell crosstalk (Figure 7M). These findings reveal significant translational potential by uncovering multiple therapeutic vulnerabilities within this signaling network. Strategies such as sphingolipid pathway blockade via SPHK1 inhibitors or FDA‐approved S1P receptor modulators, stress‐activated MAPK inhibition through JNK blockade, and interference with the SDC4–PTN axis or endosomal recycling offer promising avenues for intervention [41]. Targeting this upstream metabolic driver represents a particularly actionable strategy to disrupt the pro‐invasive niche [42, 43]. By mechanistically connecting stromal metabolic activity to neural invasion, our work establishes a new paradigm to intervene in PNI in pancreatic cancer.

## 4 Experimental Section

The experimental details are reported in the Supporting Information. Antibodies, primers, and other experimental resources are listed in Tables S4 and S5.

## Author Contributions

Conceptualization, W.P., Y.Z., and B.C.; In vitro Experiments, W.P.; In vivo Experiments, W.P., M.C., H.H., L.L., J.L., H.C., Q.Z., S.C., J.J., and L.L.; Bioinformatics analysis, W.P.; Data curation and analysis, W.P., M.C., and H.H.; Clinical specimen collection, S.B., S.X., and W.C.; Original draft writing, W.P.; Draft revision, Y.Z., and R.W.; Supervision, Z.L., Y.Z., and B.C. All authors reviewed the manuscript.

## Funding

This work was supported by the National Natural Science Foundation of China, granted numbers: No. 82173318, No. 82203812, and No. 82273432.

## Ethical Statements

Animal experiments were performed according to the Guide for the Care and Use of Laboratory Animals, with the approval of the Laboratory Animal Welfare and Ethics Committee of Tongji Hospital, Huazhong University of Science and Technology (IACUC 4463; TJH‐202501008). The Ethics Committee of Tongji Hospital granted approval for all experimental protocols (TJ‐IRB202503090), and written informed consent was obtained from all patients.

## Conflicts of Interest

The authors declare no conflicts of interest.

## Supporting information




**Supporting File 1**: advs75426‐sup‐0001‐SuppMat.docx.

**Supporting File 2**: advs75426‐sup‐0002‐TablesS1‐S6.xlsx.

**Supporting File 3**: advs75426‐sup‐0003‐FiguresS1‐S10.pdf.

## Data Availability

The data that supports the findings of this study are available in the supplementary material of this article.
