# Supplementary material for: Cancer‐Associated Fibroblast‐Derived Sphingosine‐1‐Phosphate Activates a MALL–SDC4 Axis to Facilitate Perineural Invasion in Pancreatic Cancer
Source: Adv Sci (Weinh). 2026 Apr 22;13(40):e75426. doi: 10.1002/advs.75426 (PMC13335594; doi:10.1002/advs.75426)
Supplement: Supplementary file 1 — Supporting File 1: advs75426‐sup‐0001‐SuppMat.docx. [file ADVS-13-e75426-s002.docx]

**Supporting Information**

**Cancer-associated fibroblast-derived sphingosine-1-phosphate activates a MALL-SDC4 axis to facilitate perineural invasion in pancreatic cancer**

*Wang Peng*, *Mengdie Cao*, *Hai Huang*, *Shuya Bai*, *Luyao Liu*, *Jingwen Liang*, *Haochen Cui*, *Qiaodan Zhou*, *Shiru Chen*, *Jiamei Jiang*, *Luoxia Liu*, *Zhou Luan*, *Wei Chen*, *Si Xiong*, *Ronghua Wang*, *Bin Cheng*,^*^ *Yuchong Zhao*^*^

W. Peng, M. Cao, H. Huang, S. Bai, L. Liu, J. Liang, H. Cui, Q. Zhou, S. Chen, J. Jiang, W. Chen, S. Xiong, B. Cheng, Y. Zhao Department of Gastroenterology and Hepatology, Tongji Hospital, Tongji Medical College, Huazhong University of Science and Technology, Wuhan 430030, China. E-mail: [b.cheng@tjh.tjmu.edu.cn](mailto:b.cheng@tjh.tjmu.edu.cn); [zhaoyuchong@tjh.tjmu.edu.cn](mailto:zhaoyuchong@tjh.tjmu.edu.cn)

L. Liu Department of Nuclear Medicine, Tongji Hospital, Tongji Medical College, Huazhong University of Science and Technology, Wuhan 430030, China

Z. Luan Department of Gastroenterology, Shandong Provincial Hospital Affiliated to Shandong First Medical University, Jinan 250021, China.

R. Wang Department of Internal Medicine, University of Pittsburgh Medical Center Mercy Hospital, Pittsburgh, Pennsylvania, USA

**MATERIALS AND METHODS**

Public Multi-omics Data

(1) Human pancreatic ductal adenocarcinoma (PDAC) scRNA-seq datasets were obtained from two sources: 1) Dataset CRA001160 from the Genome Sequence Archive (https://ngdc.cncb.ac.cn/gsa/), which contains clinical perineural invasion (PNI) status annotation, enabling investigation of differential cell type abundance between PNI-positive (n = 18) and PNI-negative (n = 6) tumors. 2) The Human Tumor Atlas Network (HTAN) database (https://data.humantumoratlas.org/), from which we selected samples containing Schwann cells (60 samples from 18 patients) to derive cell type signatures for subsequent deconvolution analyses in spatial and bulk transcriptomic data;^1–3^ Detailed sample information is available in the database and original literature, with a summary provided in Table S1. scRNA-seq data were analyzed using the Seurat pipeline in R version 4.4.0. Samples were integrated using standard Harmony pipeline, and cells were annotated manually by checking the expression of canonical cell type markers.

(2) Murine KPC (*Kras*^LSL‐G12D/+^; *Trp53*^LSL‐R172H/+^; *Pdx1*‐Cre) tumor scRNA-seq datasets were obtained from the Gene Expression Omnibus (GEO, https://www.ncbi.nlm.nih.gov/geo/) database (GSE114417, GSE125588, GSE156438, GSE180859, GSE202651, GSE217846, GSE247072, GSE256025, GSE165534, GSE198815, GSE129455) to analyze sphingosine kinase 1 (SPHK1) expression in cancer-associated fibroblast (CAF) subtypes. scRNA-seq data were analyzed using the Seurat pipeline in R version 4.4.0. Samples were integrated using standard Harmony pipeline, and cells were annotated manually by checking the expression of canonical cell type markers.

(3) PDAC spatial transcriptomic datasets were obtained from the GEO database (GSE235315 and GSE278687). Spatial transcriptomic data were analyzed using the Seurat pipeline in R version 4.4.0.

(4) PDAC bulk RNA-seq data with survival data were obtained from The Cancer Genome Atlas (TCGA) database (https://portal.gdc.cancer.gov/).

(5) Schwann cell bulk RNA-seq data were obtained from the ArrayExpress database (E-MTAB-6138, https://www.ebi.ac.uk/arrayexpress), and the GEO database (GSE236735, GSE133716, GSE133716, GSE171557, GSE126040, GSE59125, GSE218436, GSE141437, GSE90711, GSE153199).

Subtype Classification of CAF Subclusters

To evaluate the phenotypic states of CAFs, we calculated expression module scores for three distinct CAF subtypes: myofibroblastic CAFs (myCAFs), inflammatory CAFs (iCAFs), and antigen-presenting CAFs (apCAFs). The myCAF scoring signature included ACTA2, TAGLN, MYL9, TPM2, MMP11, MMP13, MFAP5, PDGFRB, HOPX, POSTN, COL1A1, COL1A2, COL3A1, COL5A1, COL5A2, COL11A1, COL11A2, FN1, THBS1, THBS2, LRRC15, SPARC, FAP, and ITGA11. The iCAF module score was calculated based on the expression of IL6, CXCL12, CXCL1, CXCL2, CCL2, CCL7, LIF, IL11, PDGFRA, CFD, PLA2G2A, HAS1, CLU, EMP1, LMNA, DUSP1, SOCS3, PTGS2, and DPT. The apCAF module score was derived from the expression of CD74, HLA-DRA, HLA-DRB1, HLA-DRB5, HLA-DPA1, HLA-DPB1, HLA-DQA1, HLA-DQB1, HLA-DMA, HLA-DMB, and CIITA. Module scores were computed using the AddModuleScore function in Seurat package.

Patient Samples

PDAC tissue microarrays (HPanA125Su01) were acquired from Shanghai Outdo Biotech Company (Shanghai, China) with approval from the China Human Genetic Resources Management Office. Surgical specimens for primary CAF isolation and related experiments were collected from 16 PDAC patients receiving curative surgical treatment at Tongji Hospital, Huazhong University of Science and Technology (Wuhan, China). The Ethics Committee of Tongji Hospital granted approval for all experimental protocols (TJ-IRB202503090), and written informed consent was obtained from all patients.

Cell Culture

Human pancreatic cancer cell lines AsPC-1 (RRID: CVCL_0152) and SW1990 (RRID: CVCL_1723) were obtained from the American Type Culture Collection (ATCC, Manassas, VA, USA). The immortalized human normal pancreatic duct epithelial HPDE6c7 cells (RRID: CVCL_0P38) were obtained from the Meisen Cell Technology Co., Ltd. (Zhejiang, China). The human Schwann cell line sNF96.2 (RRID: CVCL_K281) was purchased from Wuhan SAIOS Biotechnology Co., Ltd. (Wuhan, China). The human embryonic kidney cell line HEK293T (RRID: CVCL_0063) was obtained from the Cell Resource Center, Institute of Biochemistry and Cell Biology, Chinese Academy of Sciences (Shanghai, China). The mouse pancreatic cancer cell line Panc02 (RRID: CVCL_D627) was purchased from Boster Biological Technology (Wuhan, China). Primary CAFs were isolated from fresh pancreatic cancer tissues obtained from PDAC patients and KPC mice as previously described.^4^ HPDE6c7 Cells were maintained in Keratinocyte Serum-Free Medium (K-SFM) supplemented with 5 ng/mL epidermal growth factor and 50 μg/mL bovine pituitary extract. AsPC-1 cells were cultured in RPMI 1640 medium, and other cells in DMEM medium, with 10% fetal bovine serum (FBS). All media were supplemented with 1% penicillin/streptomycin (P/S). Cells were cultured at 37°C in a humidified atmosphere containing 5% CO₂. All cells were tested negative for mycoplasma contamination using the MycoBlue Mycoplasma Detector (Vazyme, Nanjing, China).

Pharmacological Inhibitors

The pharmacological inhibitors used in this study are as follows: EGFR inhibitor Gefitinib (5 μM; HY-50895, MCE), NF-κB inhibitor BAY 11-7082 (1 μM; HY-13453, MCE), JAK/STAT inhibitor Ruxolitinib (0.5 μM; HY-50856, MCE), JNK inhibitor JNK-IN-8 (1 μM; HY-13319, MCE), p38 MAPK inhibitor Doramapimod (0.5 μM; HY-10320, MCE), ERK inhibitor SCH772984 (0.1 μM; HY-50846, MCE), S1PR3 receptor antagonist TY-52156 (5 μM; HY-19736, MCE), ROCK inhibitor Y-27632 (10 μM; HY-10071, MCE) was used to block RhoA downstream signaling. Stock solutions were prepared in dimethyl sulfoxide (DMSO) or sterile water according to the manufacturer's instructions, and were diluted immediately before use. Cells were treated with the respective inhibitors for 24 hours.

Animal Experiments

Four-week-old male C57BL/6 mice were obtained from GemPharmatech Co., Ltd. (Jiangsu, China) and maintained in the Laboratory Animal Center of Tongji Hospital under specific pathogen-free conditions. Animal experiments were performed according to the Guide for the Care and Use of Laboratory Animals, with the approval of the Laboratory Animal Welfare and Ethics Committee of Tongji Hospital, Huazhong University of Science and Technology (IACUC 4463; TJH‐202501008). All animals were randomized before tumor inoculation. The sciatic nerve invasion model was conducted as described previously.^5,6^ Briefly, after gene expression modulation of cells, 5 × 10^5 Panc02 cells labeled with luciferase were inoculated alone or co-injected with CAFs (1:3 ratio) in a phosphate-buffered saline (PBS)  + Matrigel mixture to the periphery of the sciatic nerve of the right hind limb of mice. The incision was sutured aseptically. Limb function and sciatic nerve function were monitored every 3 days. Limb function was graded according to the hind limb paw response to manual extension of the body, from 4 (normal) to 1 (total paw paralysis). The sciatic function index was calculated as the spread length (mm) between the first and fifth toes of the mouse hind limbs after full extension. After 4 weeks, tumor volume was quantified using an IVIS (in vivo imaging system) imaging system following intraperitoneal administration of 200 μL of D‐Luciferin potassium (15 mg/mL; HY‐12591, MCE) dissolved in PBS. The parental mice of KPC mice were introduced and bred in the Laboratory Animal Center of Tongji Hospital under specific pathogen‐free conditions as described previously.^4^ The genotype of KPC mice was confirmed before formal experiments. ^68^Ga-DOTA-FAPI positron emission tomography/computed tomography (PET/CT) imaging was used to validate tumor formation and size in KPC mice as described previously.^7,8^ ^68^Ga-DOTA-FAPI uptake was quantified by drawing regions of interest using IRIS PET/CT software and plotting maximum uptake values (SUVmax). Mice from the same cohort were euthanized at the endpoint to collect tumor tissues for further analyses.

Immunofluorescence Staining

Cells were fixed with 4% paraformaldehyde for 10 minutes at room temperature. After fixation, cells were washed three times with PBS and permeabilized with 0.1% Triton X-100 for 10 minutes. Cells were washed three times with PBS and blocked with 5% bovine serum albumin for 1 hour. Primary antibodies were incubated overnight at 4°C. After washing three times with PBS, secondary antibodies were incubated for 1 hour at room temperature in the dark. Cells were washed three times with PBS, followed by DAPI (4′,6-diamidino-2-phenylindole) staining for 5 minutes at room temperature in the dark. After washing five times with PBS, cells were mounted and observed under fluorescence microscopy. Primary and secondary antibodies are listed in Table S4. Actin-Tracker Green-488 (C2201S, Beyotime) was used for F-actin staining.

Multiplex Immunofluorescence (mIF) Staining

PDAC tissue microarrays and wax blocks of mouse KPC tumor tissues underwent mIF analysis at Wuhan Human Genetic Resource Bank (Wuhan Biobank Co., LTD) following established protocols.^4^ Tissue sections were processed through standard deparaffinization, peroxidase inactivation, and thermal antigen unmasking procedures. Multiple rounds of antibody labeling, imaging, and signal removal were executed using specific primary antibodies in conjunction with the Opal Polaris 7 Color IHC Detection Kit (NEL871001KT, AKOYA). Image acquisition was conducted via PhenoImager HT System integrated with inForm 2.6 analytical software. Cell identification was achieved through automated nuclear detection algorithms utilizing DAPI counterstaining. Expression intensity and cellular coordinates were subsequently determined for each detected cell.

Western Blot

Western blot was performed as established procedures.^9^ For membrane protein sample preparation, denaturation was performed at room temperature for 10 min. Primary antibodies and second antibodies are listed in Table S4.

Cell Preference Analysis

To comprehensively assess differential cell type abundance between PNI-positive and PNI-negative tumors in CRA001160 dataset, we employed multiple complementary analytical approaches:

(1) Cell Ratio Analysis: Overall cell type distributions were assessed using percentage stacked bar charts, where each cell type's contribution to the total cellular composition was calculated across all samples within each PNI group. This approach provided a global view of cellular ecosystem shifts associated with PNI status.

(2) Cell Frequency Analysis: Cell type frequencies were calculated on a per-sample basis by determining the proportion of each cell type relative to the total cell count within individual samples. For each sample, cell frequencies were computed as: frequency = (cell type count / total sample cell count) × 100. Statistical comparisons between PNI groups were performed using Student's t-test.

(3) Odds Ratio (OR) Analysis: To quantify cell type-specific enrichment patterns, we calculated odds ratios for each cell type between PNI-positive and PNI-negative tumors using Fisher's exact test on 2×2 contingency tables. For each cell type-condition combination, contingency tables were constructed with the target cell type versus all other cell types across PNI conditions. An OR > 1.5 with Benjamini-Hochberg corrected P < 0.05 indicated significant enrichment in PNI-positive tumors, while an OR < 0.67 (reciprocal of 1.5) indicated significant depletion.

(4) Observed-to-Expected Ratio (Ro/e): The observed-to-expected ratio was calculated using the chi-square test framework to assess cell type distribution patterns between PNI groups. Contingency tables were constructed, and expected frequencies were computed under the assumption of independence between cell type and PNI status. The Ro/e was calculated as the ratio of observed cell counts to expected cell counts (Ro/e = observed/expected) for each cell type-condition combination. Ro/e values > 1 indicated over-representation, while values < 1 indicated under-representation relative to the null hypothesis of equal distribution. To manage extreme values, upper and lower thresholds were applied to prevent extreme ratios from dominating the visualization. Values were categorized as: Ro/e > 1 ("+++"), 0.8 < Ro/e ≤ 1 ("++"), 0.2 ≤ Ro/e ≤ 0.8 ("+"), 0 < Ro/e < 0.2 ("+/−"), and Ro/e = 0 ("−").

(5) MiloR Differential Abundance Analysis: We utilized MiloR, a robust computational approach for identifying differential cell abundance through the assignment of cells into overlapping local neighborhoods within a k-nearest neighbor network.^10^ This method identifies cell populations showing abundance differences across conditions by quantifying cell counts within neighborhoods defined according to gene expression similarity. This neighborhood-based approach uses partially overlapping neighborhoods rather than discrete clusters, allowing for more nuanced detection of abundance changes and reducing the impact of arbitrary clustering decisions on differential abundance results.

Survival and Scissor Analysis

Survival analyses were performed at both gene and cell levels using bulk RNA-seq data. Patients were stratified into high and low groups according to individual gene expression or cell type signature scores (e.g. CAF and Schwann cell signature scores) using median values. Refined cell type signatures were derived from the HTAN scRNA-seq data using the scran package with filtering criteria of summary.logFC > 1 and false discovery rate (FDR) < 0.05. Cell type signature scores were calculated on bulk RNA-seq data using the IOBR package with the principal component analysis (PCA) method by extracting the first principal component (PC1) based on the expression of cell type signature genes. Kaplan-Meier survival curves were analyzed using the log-rank test.

To identify single-cell populations associated with overall survival (OS) and disease-free survival (DFS), we also employed the Scissor algorithm using TCGA bulk RNA-seq data with survival data and CRA001160 scRNA-seq data. Scissor integrates phenotypes into a network regularized sparse regression model by linking gene expression and cell expression in bulk samples.^11^ The algorithm identifies Scissor-positive cells associated with worse patient survival and Scissor-negative cells associated with better survival outcomes.

Cell2location Spatial Deconvolution Analysis

We applied the Cell2location algorithm to resolve cell type abundance for each spot in the spatial transcriptomic data.^12^ Cell type signatures were derived from the HTAN scRNA-seq dataset, which was selected because it contained annotated Schwann cells, and the negative binomial regression model was trained using default parameters. The Cell2location model was subsequently applied with N = 20 expected cells per spot and alpha = 20 for relaxed regularization to estimate spatial cell type distributions.

Conditioned Medium Preparation

When cells reached 80% confluence, they were washed three times with PBS and cultured in fresh medium (1% FBS) for 24 hours. The culture medium was collected and filtered through a 0.22 μm membrane filter to remove cell debris. The resulting supernatant (termed conditioned medium, CM) was stored at -80°C and used for experiments within 2 weeks.

Transwell Co-culture Invasion Assay

For invasion assays, SW1990 or AsPC-1 cells (1×10⁵ cells per well) with specific treatments or gene expression modifications were suspended in serum-free medium. Cells were then seeded into the upper chambers of Transwell inserts (8-μm pore size, Corning) that had been pre-coated with Matrigel (Corning) diluted 1:8 in serum-free medium. Unless otherwise specified, human Schwann cells (sNF96.2) were cultured in the lower chambers. After 48 hours of incubation, non-invasive cells remaining on the upper surface of the membrane were removed with cotton swabs, and invaded cells on the lower surface were fixed and stained with 0.1% crystal violet solution (C0121, Beyotime). Pancreatic cancer cells were stimulated with sphingosine-1-phosphate (S1P) (5 μM; HY-108496, MCE) in medium containing 1% FBS, to reduce serum interference while preserving cell viability, for 24 h before being added to the upper chamber for the indicated assays. Recombinant pleiotrophin (PTN) protein (100 ng/ml; HY-P71907, MCE) was added to the lower chamber in indicated experiments. Images were captured, and the number of invaded cells was quantified by counting cells in five random fields per membrane. Each experiment was performed in triplicate, and results were expressed as the mean number of invaded cells per field.

Quantitative Real-Time PCR (qPCR)

Total RNA was extracted from cells using TRIzol Reagent (9108, Takara), followed by reverse transcription to synthesize the complementary DNA (cDNA) using HiScript III RT SuperMix (Vazyme, Nanjing, China). qPCR was performed using the ChamQ Universal SYBR qPCR Master Mix (Vazyme, Nanjing, China). The primers used for qPCR are listed in Table S5.

Plasmid Construction, Cell Transfection, Virus Packaging, and Infection

For knockdown assays, short hairpin RNAs (shRNAs) were individually cloned into the pLKO.1-puro vector. For overexpression assays, cDNA of each target was synthesized and cloned into the PHAGE-puro vector with a FLAG or HA label. For co-immunoprecipitation (Co-IP) assays, domain deletion mutant plasmids were constructed by Shanghai Sangon Biotech Co., Ltd.. The primers employed for plasmid construction are listed in Table S5.

For transient transfection, plasmids were transfected into HEK293T cells using Polyethylenimine Linear (PEI) MW40000 (40816ES02, YEASEN) or other cells using Lipofectamine 3000 (Invitrogen), following the manufacturer's instructions. For stable transfection, packaging plasmid psPAX2, envelope plasmid pMD2.G, and targeting plasmids were co-transfected into HEK293T cells. The viral supernatant was collected, filtered through a 0.45-µm-diameter pore (Millipore, USA), and added to the cell culture medium supplemented with polybrene (Yeasen, Shanghai, China). Stably transfected cells were selected using puromycin (ST551, Beyotime) at 5 μg/mL concentration. The lentiviral vectors overexpressing MALL (carrying a Blasticidin resistance gene) were constructed and purchased from Shanghai DesignGene Biotechnology Co., Ltd. (Shanghai, China). Stable MALL-overexpressing cells were selected using Blasticidin (ST018, Beyotime) at 10 μg/mL concentration.

Enzyme-linked immunosorbent assay (ELISA)

Conditioned media were collected and centrifuged at 1500 rpm for 10 min. S1P secretion levels were then measured using the ELISA kit (CEG031Ge, Cloud-Clone Corp.) following the manufacturer’s instructions.

mRNA-sequencing

To examine S1P-induced transcriptional changes, SW1990 cells were cultured in medium supplemented with 1% fetal bovine serum (FBS) during sphingosine-1-phosphate (S1P) treatment (5 μM; HY-108496, MCE) for 24 h. This reduced-serum condition was specifically chosen instead of the conventional complete growth medium (10% FBS) to minimize background interference noise from abundant serum-derived growth factors, cytokines, and lipid mediators. This approach preserves baseline cell viability while reducing potential masking of S1P-specific transcriptional alterations, thereby enabling a clearer and more specific evaluation of S1P-induced differential gene expression profiles. Total RNA was extracted using TRIzol Reagent (9108, Takara), and the samples were then sent to APExBIO Technology (Shanghai, China) for mRNA purification, library preparation, and paired-end mRNA sequencing. Filtered RNA-seq reads were aligned to the reference genome (GRCh38) using HISAT2, and gene expression levels were quantified using StringTie software. The DESeq2 package was used to analyze differential gene expression.

Functional Enrichment Analysis

Functional annotation and pathway enrichment analyses were performed using clusterProfiler. Differentially expressed genes were subjected to Gene Ontology (GO) biological process and Kyoto Encyclopedia of Genes and Genomes (KEGG) pathway analyses. Gene set enrichment analysis (GSEA) was performed and visualized using the clusterProfiler, msigdbr, and GseaVis packages.

RhoA-GTP Pulldown Assay

Protein lysates from cells in 6-cm dishes were divided into two portions, with half of the protein lysates separated for the determination of total RhoA levels. The remaining protein lysate was incubated with Rhotekin-RBD Agarose for 2 hours (P2065S, Beyotime). Agarose beads were collected by centrifugation, washed, and resuspended in loading buffer. RhoA levels were then detected by Western blot.

Chromatin Immunoprecipitation-qPCR (ChIP-qPCR)

A commercial ChIP assay kit (BOLG2309, BIOLOGY) was used following the manufacturer's instructions. Briefly, cells were fixed with 1% formaldehyde for 10 minutes and fragmented by sonication to shear the chromatin to 100–500 bp. The sheared crosslinked chromatin was incubated with IgG or anti-JUN (24909-1-AP, Proteintech) antibody overnight, followed by Protein A/G magnetic beads (HY-K0202, MCE) incubation. The precipitated DNA was subjected to qPCR analysis. The primers are listed in Table S5.

Immunoprecipitation-Mass Spectrometry (IP-MS) and Co-IP

In the immunoprecipitation procedure, cells were lysed using NP40 lysis buffer supplemented with protease and phosphatase inhibitors. The cell lysates were then incubated with the indicated primary antibody overnight at 4°C. The following day, Protein A/G magnetic beads (HY-K0202, MCE) were washed three times with 0.5% PBST (PBS with 0.05% Tween-20) and then added to the antibody–lysate mixture. After a 4-hour incubation at 4°C with gentle rotation, the beads were washed six times to remove non-specifically bound proteins. For the IP-MS assay, cell lysates of SW1990 cells stably overexpressing MALL-HA were incubated with anti-HA antibody (81290-1-RR, Proteintech), and the harvested beads were sent to Beijing Qinglian Biotech Co., Ltd. for subsequent mass spectrometry analysis. For the Co-IP assays, the harvested beads were eluted with loading buffer and denatured at room temperature for 10 minutes for Western blot analysis. Primary and secondary antibodies are listed in Table S4.

Lipid Raft Disruption Assay and Co-IP

To deplete membrane cholesterol and disrupt lipid raft microdomains, cells grown to 70-80% confluence were washed twice with cold PBS and incubated with methyl-β-cyclodextrin (MβCD; HY-101461, MCE), a cholesterol-depleting agent that selectively disrupts lipid raft microdomains, at a concentration of 5 mM in serum-free medium at 37°C for 40 minutes. MβCD selectively extracts cholesterol from the plasma membrane, thereby disrupting the structural integrity of lipid raft microdomains. Control cells were incubated in parallel with serum-free medium. Following MβCD treatment, cells were washed three times with ice-cold PBS and immediately subjected to Co-IP assay as described above.

Molecular docking

To explore the interactions between MALL (UniProt accession: Q13021) and SDC4 (UniProt accession: P31431), computational docking simulations were conducted. Structural models for both proteins were retrieved from the AlphaFold Protein Structure Database (https://alphafold.ebi.ac.uk/). Docking calculations were executed using the HDOCK SERVER platform, with SDC4 designated as the receptor molecule and MALL as the ligand molecule.^13^ The algorithm generates a "docking score," which is used by HDOCK to rank docking models, with more negative values indicating more favorable predicted binding, and a "confidence score," which is an empirical docking score-dependent metric calculated to indicate the binding likelihood of two molecules. According to the official HDOCK website (http://hdock.phys.hust.edu.cn/), the confidence score is calculated from the docking score as follows: Confidence score = 1.0/[1.0 + e^(0.02 × (Docking score + 150))]. Empirically, when the confidence score is > 0.7, the two molecules are considered very likely to bind; when the confidence score is between 0.5 and 0.7, the molecules are considered possible to bind; and when the confidence score is < 0.5, the molecules are considered unlikely to bind. Protein preparation steps, including removal of water molecules and extraneous ligands along with hydrogen atom addition, were carried out in PyMOL version 2.4. The top-ranked complex with the most favorable docking score was selected as the optimal docking conformation, and interaction visualization was generated through PyMOL.

Cycloheximide (CHX) Chase Assay

Cycloheximide chase analyses were performed in two experimental setups. Experiment 1 quantified SDC4 half-life in control versus MALL-overexpressing cells by adding CHX (100 µg/mL; HY-12320, MCE) and harvesting at 0, 4, 8, and 12 hours. Experiment 2 assessed degradation pathways by exposing parallel plates to CHX plus either vehicle, MG132 (10 µM; HY-13259, MCE), or bafilomycin A1 (Baf A1, 200 nM; HY-100558, MCE) and sampling at the same time points. For Experiment 2, MG132 or Baf A1 was pre-added 30 minutes before CHX and left in the medium throughout the chase. SDC4 levels were then detected by Western blot.

Flow Cytometry

Cells were harvested and resuspended in PBS containing 2% FBS. Cells were incubated with Alexa Fluor 647-conjugated anti-SDC4 antibody (A26066, ABclonal) or isotype control antibody following the manufacturer's instructions in the dark. After washing three times with PBS/2% FBS, cells were analyzed by flow cytometry. SDC4 expression was quantified as the percentage of positive cells and mean fluorescence intensity (MFI) using FlowJo software. All experiments were performed in triplicate. Cells were pre-treated with the endosomal recycling inhibitor primaquine (300 µM; HY-12651A, MCE) for 1 hour.

Cell Surface Biotinylation Assay

Cells in 6-cm dishes were washed three times with ice-cold PBS on ice to halt membrane trafficking, then surface-labeled for 30 minutes at 4 °C with 0.5 mL of 0.5 mg/mL Sulfo-NHS-SS-Biotin (A8005, APExBIO) in ice-cold PBS with gentle rocking. Excess reagent was quenched twice with 100 mM glycine in ice-cold PBS for 10 min each, followed by three ice-cold PBS washes. Cells were lysed on ice for 30 minutes in 100 µL 1% Triton X-100 in Tris-buffered saline (IP buffer) with protease inhibitors (no reducing agents). After lysis, samples were centrifuged at 15,000 × g for 5 minutes at 4 °C, and the supernatant was collected; 20 µL was reserved as input, and the remaining 80 µL was incubated with 10 µL streptavidin magnetic beads (L-1012, Biolinkedin) for 2 hours at 4 °C with rotation. Beads were washed five times with IP buffer and eluted in 30 µL loading buffer at room temperature for 10 minutes. Pulled-down biotinylated proteins and inputs were analyzed by Western blot.

Surface Biotinylation Assay for Measuring Protein Recycling

Cells were serum-starved for 4 hours and surface-labeled on ice with Sulfo-NHS-SS-Biotin (0.5 mg/mL, 30 minutes), followed by quenching with 100 mM glycine in PBS. One dish was lysed immediately to obtain the surface control (Surf0). The remaining dishes were shifted to pre-warmed culture medium and incubated at 37°C for 30 minutes to allow internalization, returned to 4 °C, and subjected to two 15-minute rounds of reducing stripping buffer (50 mM glutathione, 75 mM NaCl, 10 mM EDTA, 1% BSA, 75 mM NaOH) before lysis to yield the 30-minute internalized pool (In30). For recycling, parallel cultures after the first strip were re-warmed to 37 °C for 30 min; cells were either lysed directly (Rec60) or underwent the same second strip twice at 4 °C before lysis (S60). Cell lysates were normalized by total protein concentration using the bicinchoninic acid (BCA) assay before streptavidin pulldown. Biotinylated proteins were enriched on streptavidin beads and analyzed by Western blot. Internalization rate (%) = (In30 / Surf0) × 100. Recycling rate (%) = [(Rec60 − S60) / In30] × 100.

Statistical Analysis

Results are expressed as mean (SD) obtained from triplicate independent experiments. Spearman's rank correlation coefficient was employed for correlation assessments. The log-rank test was applied to analyze survival data. Differences across groups were assessed via Student's t-test or one-way ANOVA, with Tukey's multiple comparison test applied when necessary. Statistical significance was defined as *p* < 0.05. Significant differences are indicated by **p* < 0.05, ***p* < 0.01, and ****p* < 0.001.

# **REFERENCES**

1. Rozenblatt-Rosen, O., Regev, A., Oberdoerffer, P., Nawy, T., Hupalowska, A., Rood, J.E., Ashenberg, O., Cerami, E., Coffey, R.J., Demir, E., et al. (2020). The Human Tumor Atlas Network: Charting Tumor Transitions across Space and Time at Single-Cell Resolution. Cell *181*, 236–249. https://doi.org/10.1016/j.cell.2020.03.053.

2. de Bruijn, I., Nikolov, M., Lau, C., Clayton, A., Gibbs, D.L., Mitraka, E., Pozhidayeva, D., Lash, A., Sumer, S.O., Altreuter, J., et al. (2025). Sharing data from the Human Tumor Atlas Network through standards, infrastructure and community engagement. Nat Methods *22*, 664–671. https://doi.org/10.1038/s41592-025-02643-0.

3. Cui Zhou, D., Jayasinghe, R.G., Chen, S., Herndon, J.M., Iglesia, M.D., Navale, P., Wendl, M.C., Caravan, W., Sato, K., Storrs, E., et al. (2022). Spatially restricted drivers and transitional cell populations cooperate with the microenvironment in untreated and chemo-resistant pancreatic cancer. Nat Genet *54*, 1390–1405. https://doi.org/10.1038/s41588-022-01157-1.

4. Cao, M., Peng, W., Cheng, B., Wang, R., Chen, W., Liu, L., Huang, H., Chen, S., Cui, H., Liang, J., et al. (2025). PPY‐Induced iCAFs Cultivate an Immunosuppressive Microenvironment in Pancreatic Cancer. Advanced Science, 2413432. https://doi.org/10.1002/advs.202413432.

5. Zhang, W., He, R., Yang, W., Zhang, Y., Yuan, Q., Wang, J., Liu, Y., Chen, S., Zhang, S., Zhang, W., et al. (2022). Autophagic Schwann cells promote perineural invasion mediated by the NGF/ATG7 paracrine pathway in pancreatic cancer. J Exp Clin Cancer Res *41*, 48. https://doi.org/10.1186/s13046-021-02198-w.

6. Liu, Y., Han, G., Feng, K., Lin, X., Zhong, W., Liu, Y., Wang, C., Zhang, C., and Liu, X. (2025). ITGA5-expressing tumor cells interact with Schwann cells to drive nerve growth factor-mediated immunosuppression of NK cells. Molecular Therapy, S1525001625005799. https://doi.org/10.1016/j.ymthe.2025.07.043.

7. Chen, W., Peng, W., Wang, R., Bai, S., Cao, M., Xiong, S., Li, Y., Yang, Y., Liang, J., Liu, L., et al. (2024). Exosome-derived tRNA fragments tRF-GluCTC-0005 promotes pancreatic cancer liver metastasis by activating hepatic stellate cells. Cell Death Dis *15*, 102. https://doi.org/10.1038/s41419-024-06482-3.

8. Liu, L., Shi, Y., He, S., Yang, J., Song, S., Wang, D., Wang, Z., Zhou, H., Deng, X., Zou, S., et al. (2025). The molar dose of FAPI administered impacts on the FAP-targeted PET imaging and therapy in mouse syngeneic tumor models. Eur J Nucl Med Mol Imaging *52*, 2198–2211. https://doi.org/10.1007/s00259-025-07071-y.

9. Huang, H., Peng, W., Zhou, Q., Zhao, Y., Liu, L., Cui, H., Liang, J., Cao, M., Chen, W., Wang, R., et al. (2025). Senescent fibroblasts secrete CTHRC1 to promote cancer stemness in hepatocellular carcinoma. Cell Commun Signal *23*, 379. https://doi.org/10.1186/s12964-025-02369-8.

10. Dann, E., Henderson, N.C., Teichmann, S.A., Morgan, M.D., and Marioni, J.C. (2022). Differential abundance testing on single-cell data using k-nearest neighbor graphs. Nat Biotechnol *40*, 245–253. https://doi.org/10.1038/s41587-021-01033-z.

11. Sun, D., Guan, X., Moran, A.E., Wu, L.-Y., Qian, D.Z., Schedin, P., Dai, M.-S., Danilov, A.V., Alumkal, J.J., Adey, A.C., et al. (2022). Identifying phenotype-associated subpopulations by integrating bulk and single-cell sequencing data. Nat Biotechnol *40*, 527–538. https://doi.org/10.1038/s41587-021-01091-3.

12. Kleshchevnikov, V., Shmatko, A., Dann, E., Aivazidis, A., King, H.W., Li, T., Elmentaite, R., Lomakin, A., Kedlian, V., Gayoso, A., et al. (2022). Cell2location maps fine-grained cell types in spatial transcriptomics. Nat Biotechnol *40*, 661–671. https://doi.org/10.1038/s41587-021-01139-4.

13. Yan, Y., Tao, H., He, J., and Huang, S.-Y. (2020). The HDOCK server for integrated protein–protein docking. Nat Protoc *15*, 1829–1852. https://doi.org/10.1038/s41596-020-0312-x.
