# Supplementary material for: Cancer‐Associated Fibroblast‐Derived Sphingosine‐1‐Phosphate Activates a MALL–SDC4 Axis to Facilitate Perineural Invasion in Pancreatic Cancer
Source: Adv Sci (Weinh). 2026 Apr 22;13(40):e75426. doi: 10.1002/advs.75426 (PMC13335594; doi:10.1002/advs.75426)
Supplement: Supplementary file 3 — Supporting File 3: advs75426‐sup‐0003‐FiguresS1‐S10.pdf. [file ADVS-13-e75426-s003.pdf]

Supporting Information

Cancer-associated fibroblast-derived sphingosine-1-phosphate activates a MALL-SDC4 axis to facilitate perineural invasion in pancreatic cancer

Wang Peng, *Mengdie Cao*, *Hai Huang*, *Shuya Bai*, *Luyao Liu*, *Jingwen Liang*, *Haochen Cui*, *Qiaodan Zhou*, *Shiru Chen*, *Jiamei Jiang*, *Luoxia Liu*, *Zhou Luan*, *Wei Chen*, *Si Xiong*, *Ronghua Wang*, *Bin Cheng*,* *Yuchong Zhao**

W. Peng, M. Cao, H. Huang, S. Bai, L. Liu, J. Liang, H. Cui, Q. Zhou, S. Chen, J. Jiang, W. Chen, S. Xiong, B. Cheng, Y. Zhao Department of Gastroenterology and Hepatology, Tongji Hospital, Tongji Medical College, Huazhong University of Science and Technology, Wuhan 430030, China.

E-mail: b.cheng@tjh.tjmu.edu.cn; zhaoyuchong@tjh.tjmu.edu.cn

L. Liu Department of Nuclear Medicine, Tongji Hospital, Tongji Medical College, Huazhong University of Science and Technology, Wuhan 430030, China

Z. Luan Department of Gastroenterology, Shandong Provincial Hospital Affiliated to Shandong First Medical University, Jinan 250021, China.

R. Wang Department of Internal Medicine, University of Pittsburgh Medical Center Mercy Hospital, Pittsburgh, Pennsylvania, USA

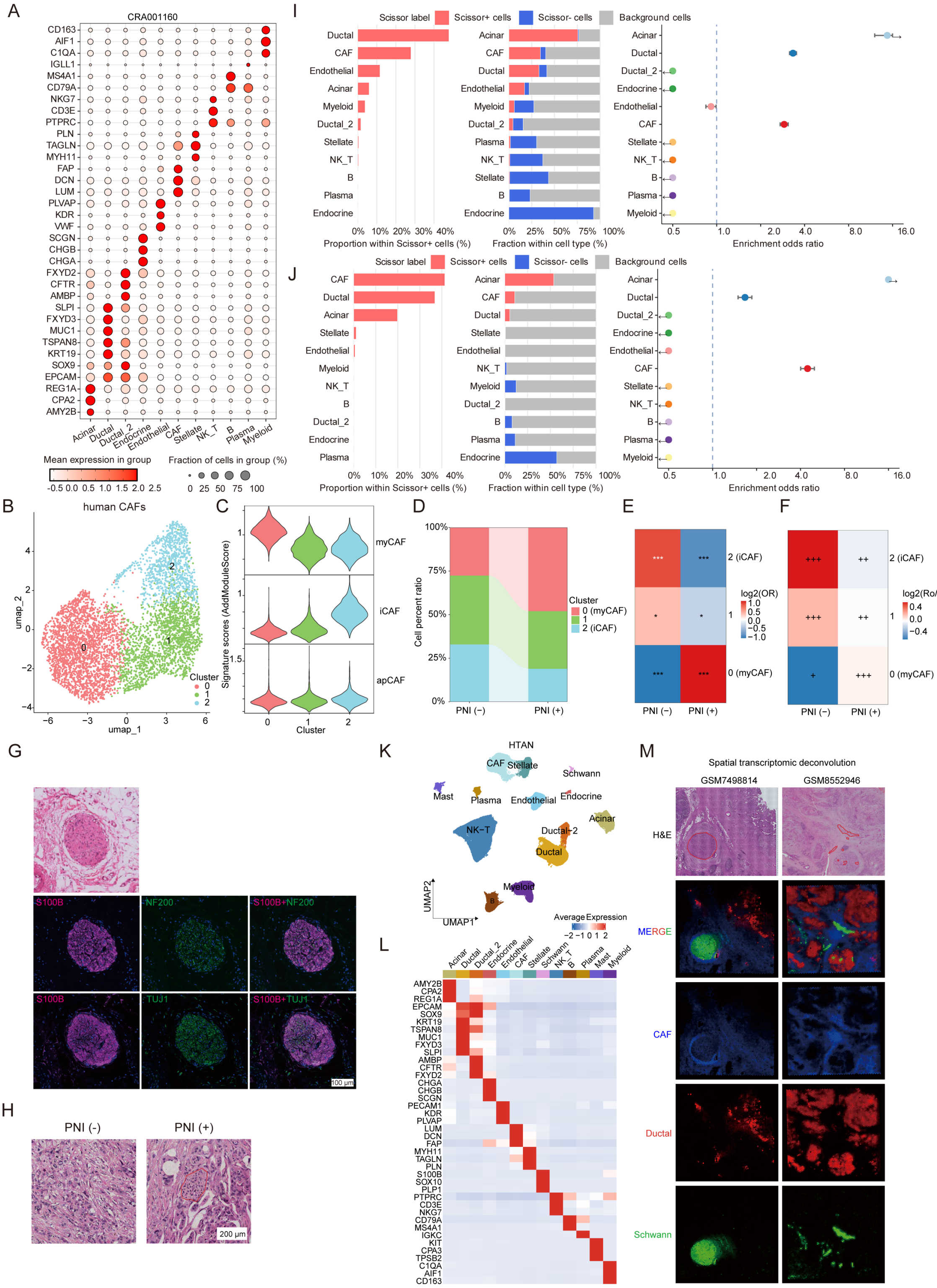

Figure S1 | Characterization of CAFs and Schwann cells in the PDAC tumor microenvironment.

A) Dot plot visualizing expression levels and fraction of representative marker genes across all

annotated cell types in the PDAC scRNA-seq dataset CRA001160. **B)** UMAP visualization of CAF subclusters in CRA001160 dataset. **C)** Violin plot showing CAF subtype signature scores calculated by AddModuleScore analysis in CAF subclusters in CRA001160 dataset. **D)** Percentage stacked bar chart displaying the proportions of CAF subclusters stratified by perineural invasion (PNI) status in CRA001160 dataset. **E)** Odds ratios (OR) of CAF subclusters according to PNI status in CRA001160 dataset. **F)** Observed-to-expected ratios (Ro/e) of CAF subclusters according to PNI status in CRA001160 dataset. **G)** Hematoxylin and eosin staining and immunofluorescence staining images demonstrating S100B-positive Schwann cells co-localizing with NF200-positive axonal structures and TUJ1-positive neuronal elements in human PDAC tissues, confirming the localization of Schwann cells along peripheral nerve fibers within the tumor microenvironment. Scale bars, 100 μm. **H)** Representative hematoxylin and eosin staining of tumor sections showing tumor cell infiltration around nerve structures. Scale bars, 200 μm. **I, J)** Scissor analysis results for overall survival (OS) **(I)** and disease-free survival (DFS) **(J)** associations: (left panel) bar charts showing the proportion of each cell type among Scissor-positive cells; (middle panel) stacked bar charts displaying the relative contributions of Scissor-positive, Scissor-negative, and Scissor-background cell fractions for each annotated cell type; (right panel) forest plots presenting enrichment odds ratios with 95% confidence intervals quantifying the strength of association between each cell type and patient survival outcomes. **K)** UMAP visualization of the HTAN PDAC scRNA-seq dataset showing distinct cell populations. **L)** Heatmap showing the average expression of canonical markers across annotated cell types in HTAN. **M)** Spatial transcriptomic maps displaying the abundance and distribution of ductal cells, Schwann cells, and CAFs deconvoluted by Cell2location algorithm in the tumor microenvironment.

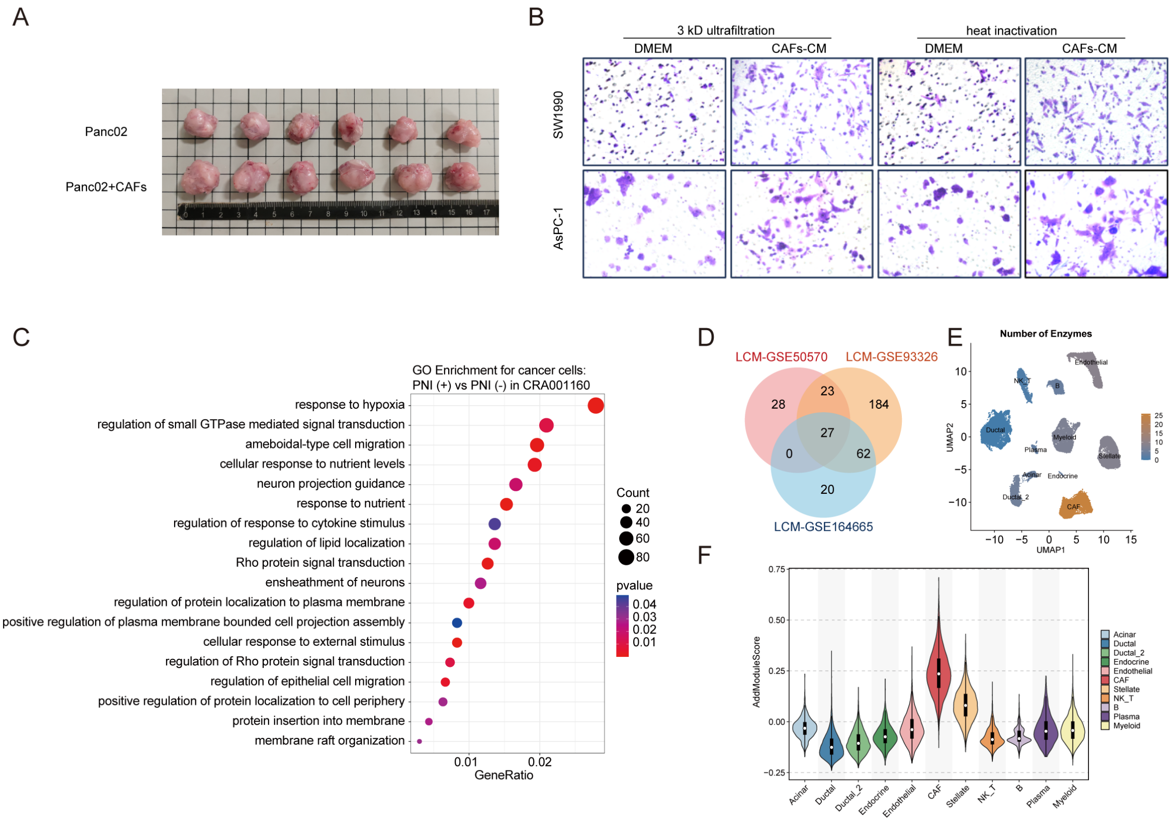

Figure S2 | Cancer-associated fibroblasts promote pancreatic cancer progression

A) Gross images of tumors in the sciatic nerve invasion model. **B)** Transwell invasion assays showing that filtered and heat-inactivated CAF-conditioned medium (CAF-CM) retains pro-invasion activity compared to DMEM and filtered or heat-inactivated DMEM controls. **C)** Gene Ontology functional enrichment analyses comparing tumor cells from PNI-positive and PNI-negative cases in the PDAC scRNA-seq dataset CRA001160. **D)** Venn diagram of three laser capture microdissection RNA sequencing datasets identifying 27 metabolic enzymes consistently upregulated in stromal regions relative to tumor regions. **E, F)** UMAP projection of the 27 identified metabolic enzymes onto the CRA001160 dataset showing predominant expression in CAFs **(E)** and the highest module scores in CAFs as determined by AddModuleScore analysis **(F)**.

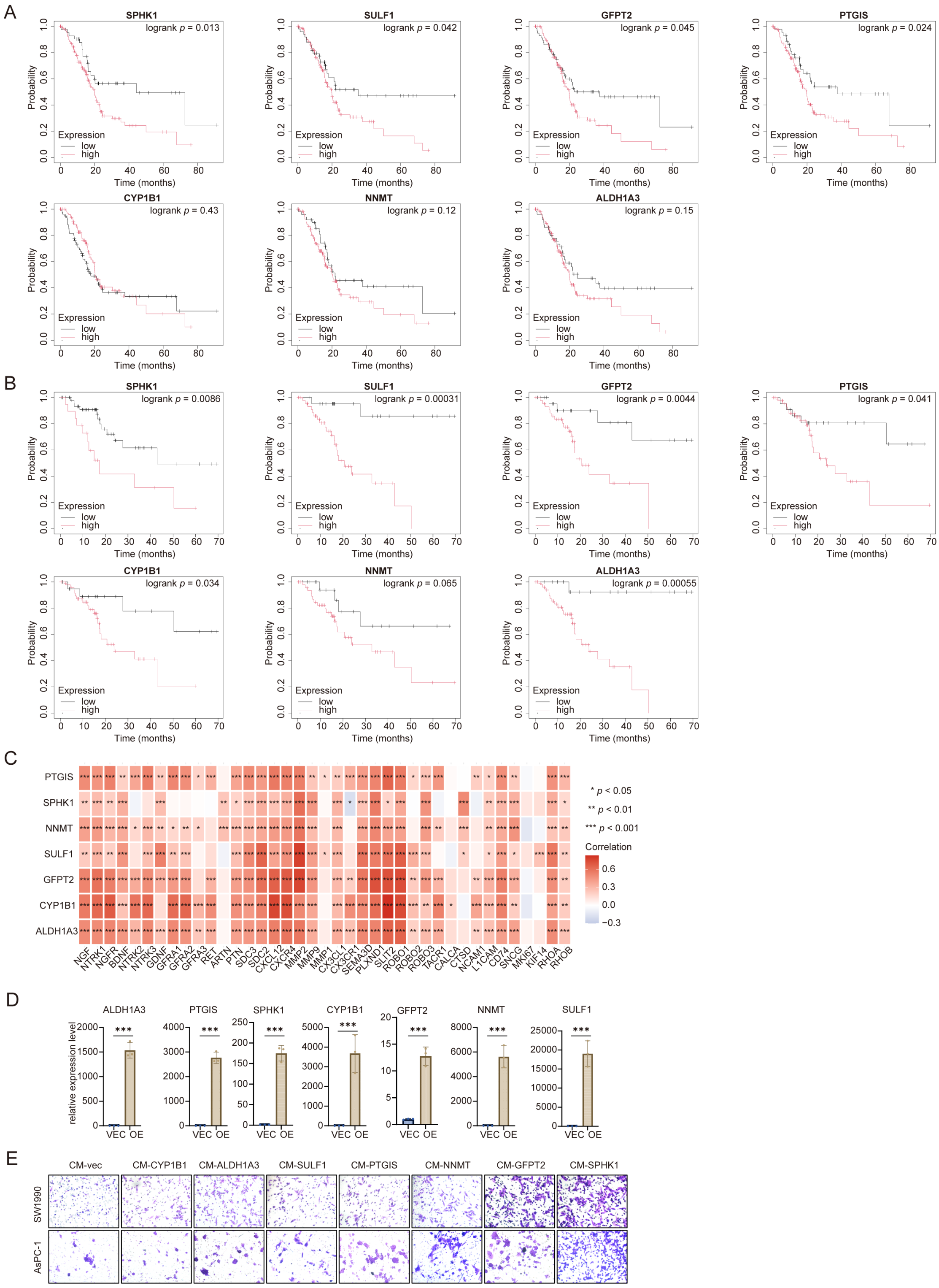

Figure S3 | Survival analysis of candidate enzymes and their correlations with perineural invasion in PDAC

A, B) Kaplan-Meier survival curves for overall survival **(A)** and disease-free survival **(B)** stratified by the expression levels of each candidate metabolic enzyme in the TCGA-PAAD dataset. **C)** Correlation heatmap showing expression associations between seven candidate metabolic enzymes and perineural invasion-related genes in the TCGA-PAAD dataset. **D)** Quantitative PCR validation confirming the overexpression efficiency of the seven candidate metabolic enzymes in HEK293T cells used for conditioned medium screening experiments. **E)** Transwell invasion assays showing differential cancer cell invasion patterns in response to conditioned media from HEK293T cells overexpressing each of the seven candidate metabolic enzymes. Correlation analyses were performed using the Spearman correlation method. Survival curves were analyzed using the log-rank test. Data are presented as mean (SD) and were analyzed using Student's t-test for two-group comparisons or one-way ANOVA followed by Tukey's post-hoc test for multiple comparisons as appropriate. *$p < 0.05$, **$p < 0.01$, ***$p < 0.001$.

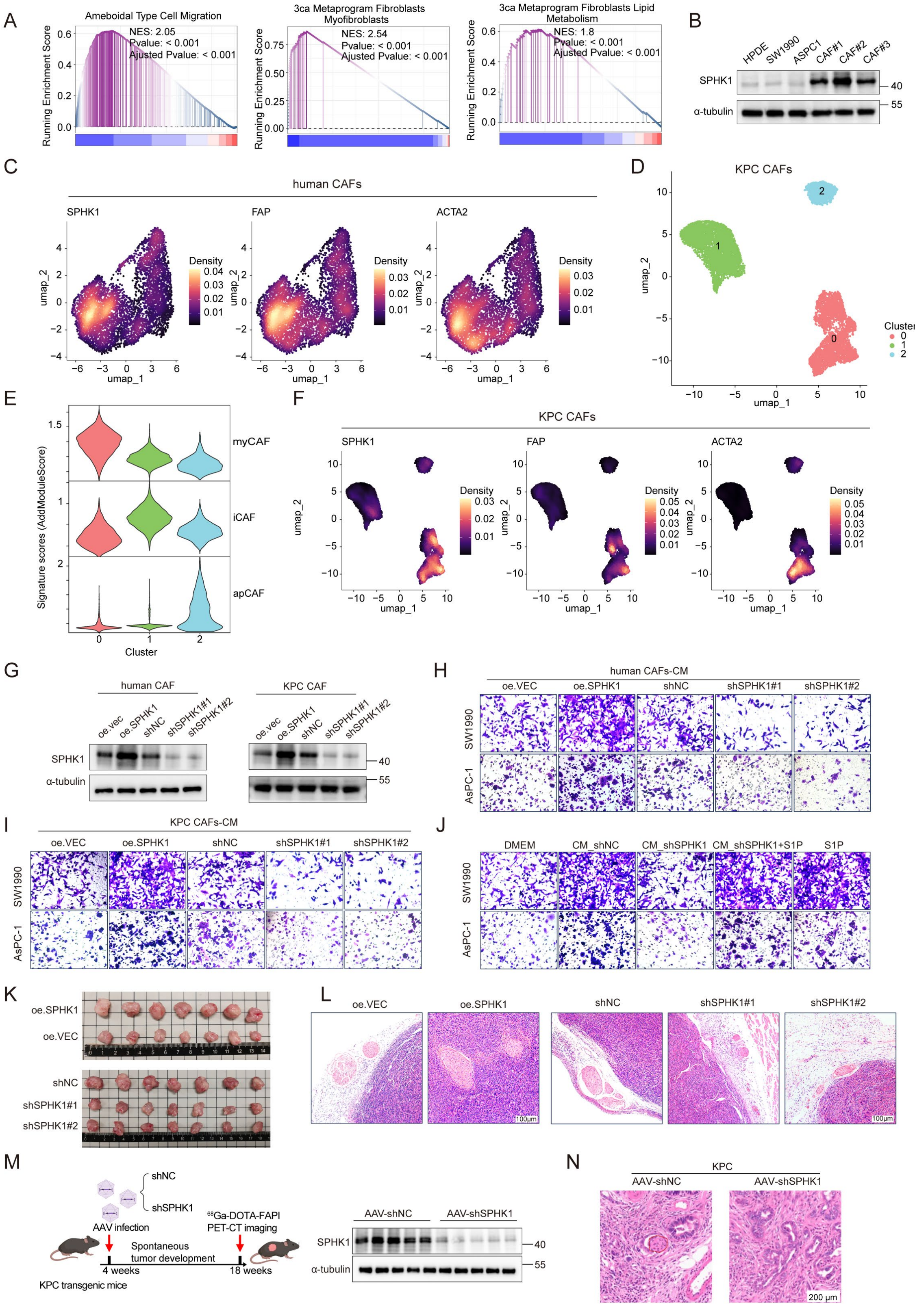

Figure S4 | SPHK1 is upregulated in cancer-associated fibroblasts and drives perineural invasion in pancreatic cancer

A) Gene Set Enrichment Analysis of TCGA-PAAD tumors with high SPHK1 expression. **B)** Western blot analysis showing SPHK1 expression in normal pancreatic HPDE6c7 cells, pancreatic cancer cell lines (SW1990 and AsPC-1), and three independent CAF isolates. **C)** UMAP plot displaying SPHK1, FAP, and ACTA2 expression across CAF subclusters in the CRA001160 dataset. **D)** UMAP visualization of CAF subclusters in the integrated scRNA-seq dataset from KPC mice. **E)** Violin plot showing CAF subtype signature scores calculated by AddModuleScore analysis in CAF subclusters from KPC mice. **F)** UMAP plot displaying SPHK1, FAP, and ACTA2 expression in CAF subclusters from KPC mice. **G)** Western blot analysis demonstrating efficient SPHK1 modulation (knockdown and overexpression) in both human primary CAFs and primary CAFs derived from KPC mice. **H-I)** Transwell invasion assays showing cancer cell invasion in response to conditioned media from human PDAC-derived CAFs **(H)** or murine KPC-derived CAFs **(I)** under SPHK1 knockdown, overexpression, or control conditions. **J)** Transwell invasion assays showing enhanced cancer cell invasion following direct S1P treatment (5 μM). **K)** Gross images of tumors in the sciatic nerve invasion model (n = 7 per group). **L)** Representative hematoxylin and eosin sections showing increased neural encasement in mice co-injected with SPHK1-overexpressing CAFs and decreased neural encasement in mice co-injected with SPHK1-knockdown CAFs, compared with controls. Scale bars, 100 μm. **M)** Adeno-associated virus (AAV)-mediated pancreatic SPHK1 knockdown in KPC mice. **N)** Representative hematoxylin and eosin staining of tumor sections showing tumor cell infiltration around nerve structures in KPC mice. Scale bars, 200 μm.

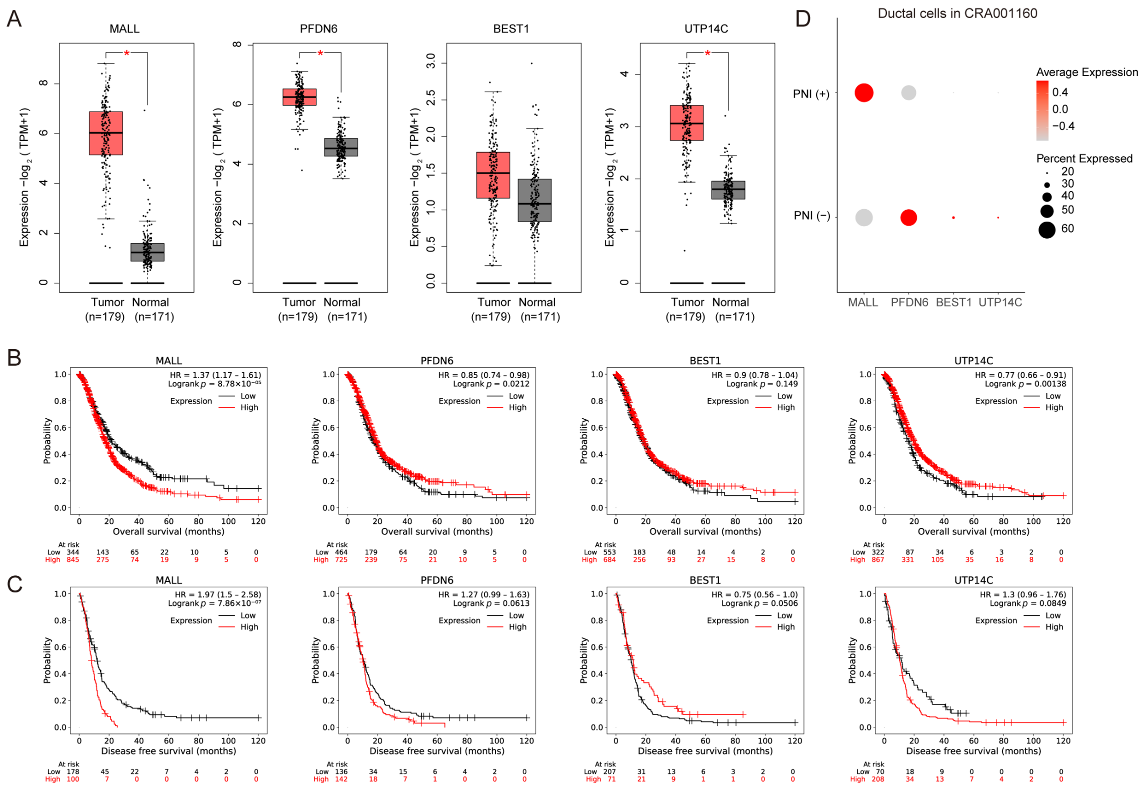

Figure S5 | Expression patterns and survival analysis of the top four S1P-upregulated genes

A) Boxplot showing the expression of four candidate genes in PDAC tumor tissues and normal pancreatic tissues, as analyzed using the GEPIA2 database (http://gepia2.cancer-pku.cn/). **B, C)** Kaplan-Meier survival curves for overall survival **(B)** and disease-free survival **(C)** stratified by the expression levels of each candidate gene, as analyzed using Kaplan-Meier Plotter (http://kmplot.com/). **D)** Dot plot visualizing the expression levels and fraction of four candidate genes in ductal cells stratified by PNI status in the CRA001160 dataset.

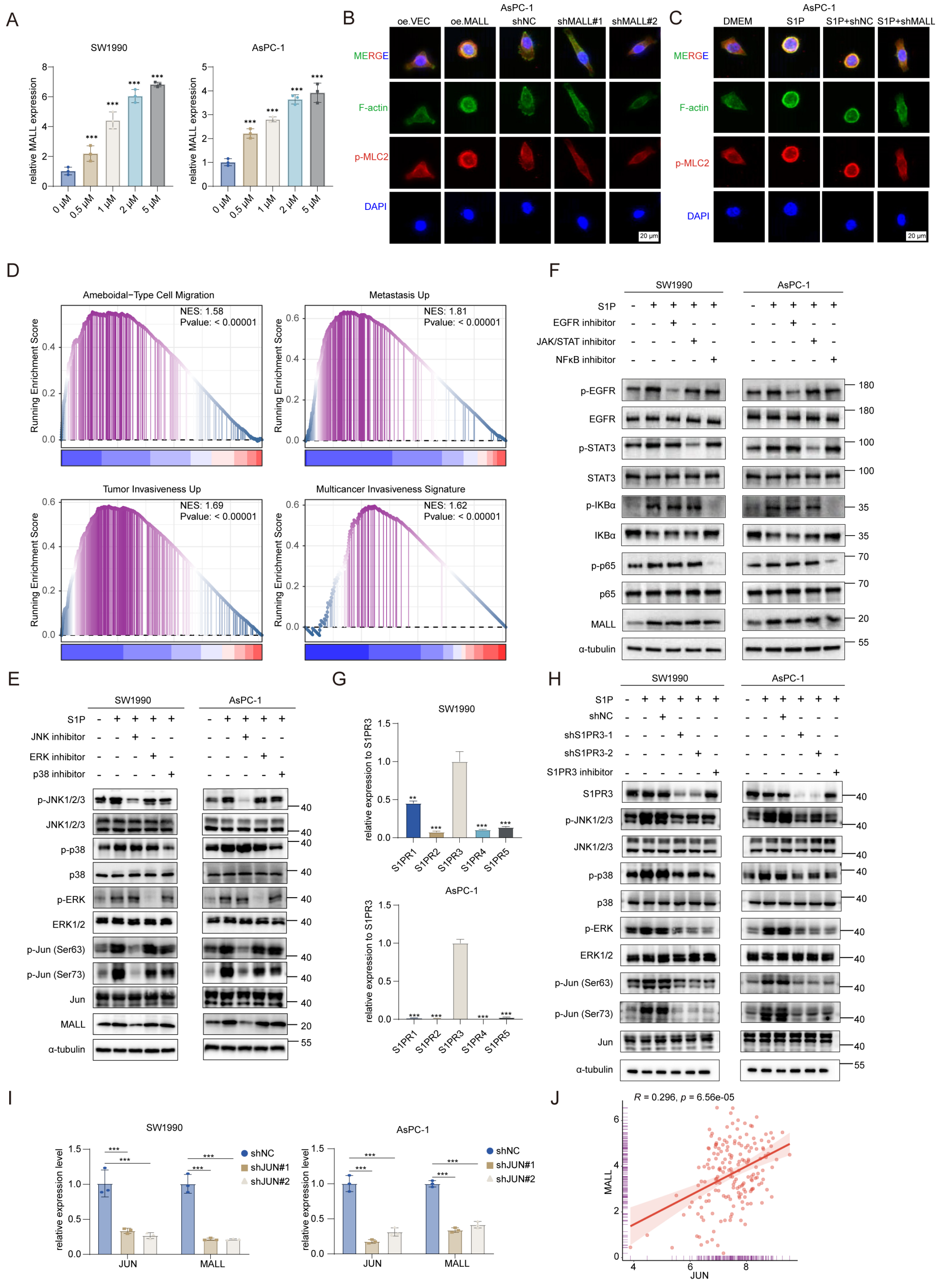

Figure S6 | Sphingosine-1-phosphate–MALL signaling axis drives amoeboid transition

A) qPCR analysis of MALL mRNA expression following treatment with different concentrations of S1P. **B)** Representative immunofluorescence images from MALL gain-of-function and loss-of-

function experiments in AsPC-1 cells. **C)** Immunofluorescence analysis showing that S1P-induced amoeboid transition occurs in a MALL-dependent manner in AsPC-1 cells. **D)** Gene Set Enrichment Analysis of TCGA-PAAD tumors with high MALL expression. **E)** Pharmacological dissection using selective MAPK inhibitors demonstrating that JNK blockade (1 μM JNK-IN-8) specifically abrogates (5 μM) S1P-induced MALL upregulation, whereas ERK (0.1 μM SCH772984) and p38 (0.5 μM Doramapimod) inhibition has limited effects. **F)** Pharmacological inhibition of the EGFR (5 μM Gefitinib), JAK/STAT (0.5 μM Ruxolitinib), or NF-κB (1 μM BAY 11-7082) pathway has limited effects on (5 μM) S1P-induced MALL upregulation. **G)** qPCR analysis determining the relative expression levels of sphingosine-1-phosphate receptors S1PR1, S1PR2, S1PR3, S1PR4, and S1PR5 in pancreatic cancer cells, highlighting S1PR3 as the predominantly expressed receptor subtype. **H)** S1PR3 knockdown and pharmacological inhibition (5 μM TY-52156) significantly diminish S1P-induced JNK activation and MALL expression, establishing S1PR3 as the key receptor mediating this response. **I)** qPCR analysis measuring MALL mRNA expression following JUN knockdown in pancreatic cancer cells. **J)** Correlation analysis between JUN and MALL mRNA expression in TCGA-PAAD tumor specimens. Correlation analyses were performed using the Spearman correlation method. Survival curves were analyzed using the log-rank test. Data are presented as mean (SD) and were analyzed using Student's t-test for two-group comparisons or one-way ANOVA followed by Tukey's post-hoc test for multiple comparisons as appropriate. *$p < 0.05$, **$p < 0.01$, ***$p < 0.001$.

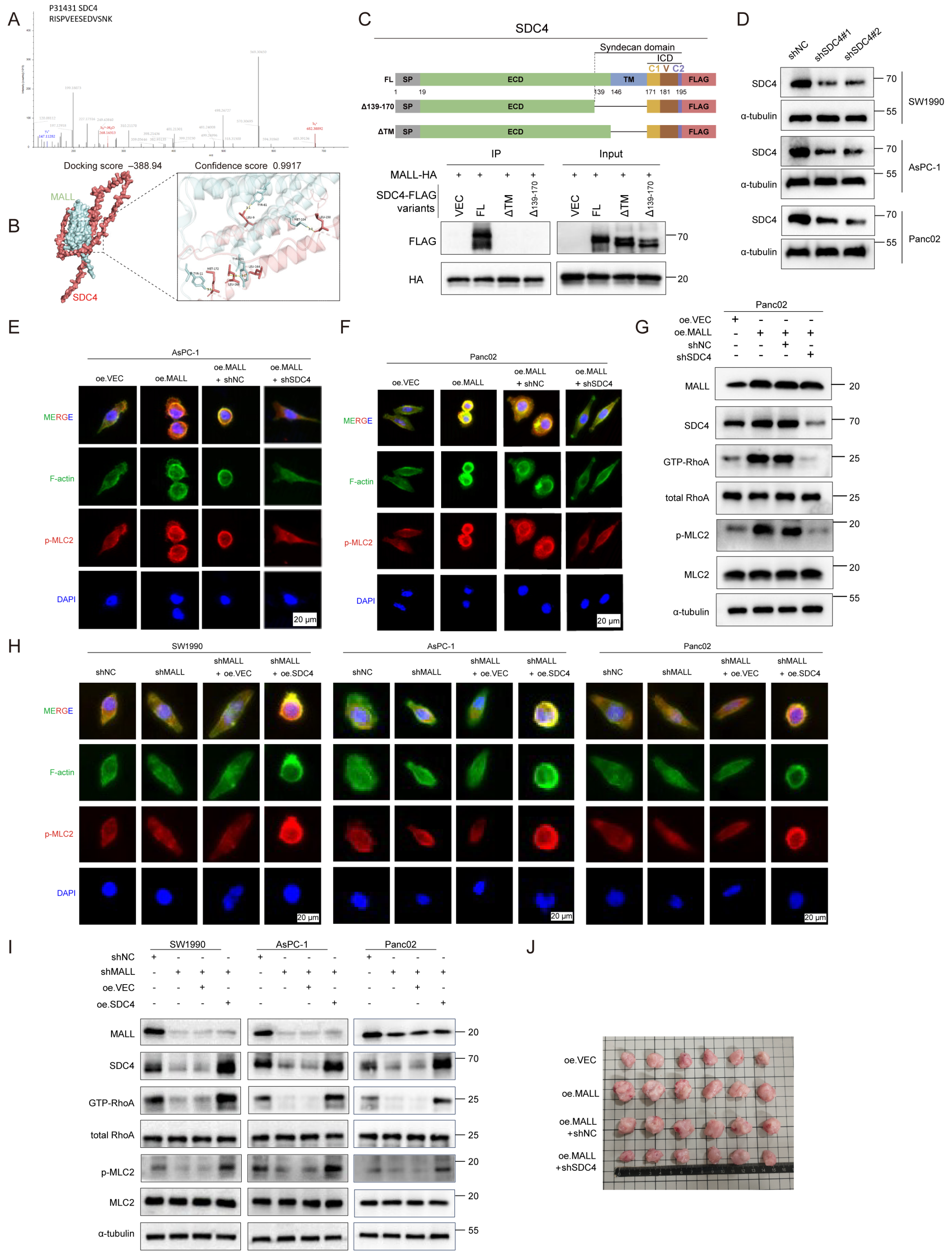

Figure S7 | MALL–SDC4 interaction is required for amoeboid transition

A) Mass spectrometry analysis of MALL co-immunoprecipitation identifying syndecan-4 (SDC4) as a major interacting partner. **B)** *In silico* molecular docking analysis supporting a plausible

MALL–SDC4 protein-protein interface, showing predicted binding conformations and interaction scores. **C)** Co-immunoprecipitation analysis using full-length MALL with SDC4 ΔTM and Δ AA139–170 deletion mutants. **D)** Western blot analysis confirming the knockdown efficiency of SDC4 in pancreatic cancer cells. **E, F)** Immunofluorescence images from sequential perturbation experiments combining MALL overexpression with SDC4 knockdown in AsPC-1 **(E)** and Panc02 **(F)** cells, showing that SDC4 loss suppresses MALL-driven amoeboid transition. **G)** Sequential perturbation experiments combining MALL overexpression with SDC4 knockdown, showing that SDC4 loss suppresses MALL-induced p-MLC2 pathway activation. **H, I)** Immunofluorescence staining **(H)** and Western blot analysis **(I)** from rescue experiments with SDC4 overexpression in MALL-knockdown cells, showing that SDC4 overexpression restores p-MLC2 signaling pathway activation and amoeboid transition suppressed by MALL knockdown. **J)** Gross images of tumors in the sciatic nerve invasion model under sequential perturbation conditions (n = 6 per group).

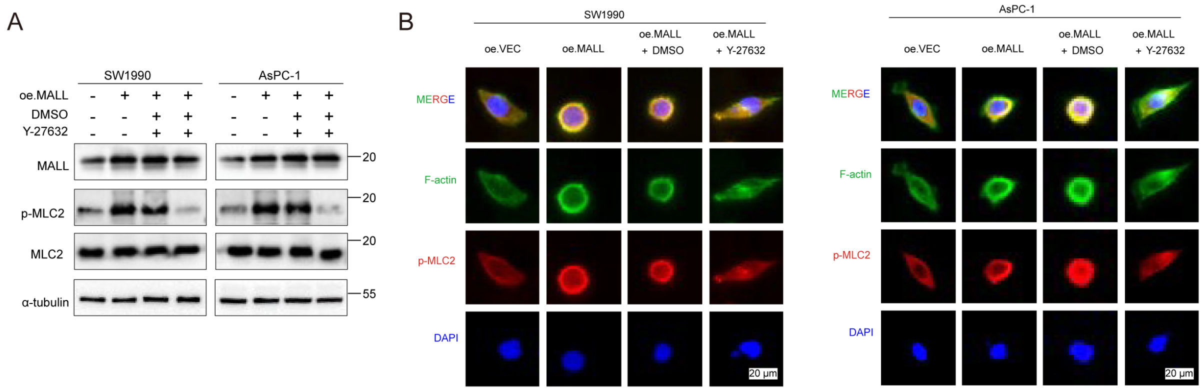

Figure S8 | MALL promotes amoeboid transition through RhoA pathway activity

A, B) Sequential perturbation experiments combining MALL overexpression with RhoA pathway inhibition by Y-27632 (10 μM) show that RhoA pathway inhibition suppresses MALL-driven amoeboid transition, as shown by Western blot analysis **(A)** and immunofluorescence images **(B)**.

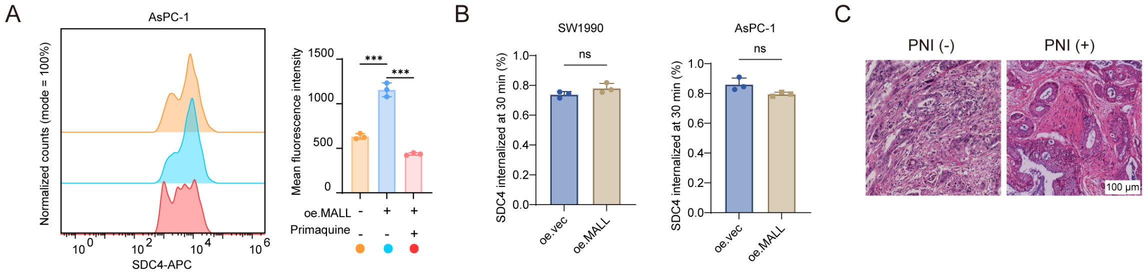

Figure S9 | MALL increases SDC4 surface levels by enhancing endosomal recycling

A) Flow cytometry demonstrates that treatment with the recycling inhibitor primaquine (300 μM) blocks the MALL-mediated increase in SDC4 surface expression in AsPC-1 cells. **B)** SDC4 internalization rate in control and MALL-overexpressing cancer cells. **C)** Representative hematoxylin and eosin staining of tumor sections showing tumor cell infiltration around nerve structures. Scale bars, 100 μm. Data are presented as mean (SD) and were analyzed using Student's t-test for two-group comparisons or one-way ANOVA followed by Tukey's post-hoc test for multiple comparisons as appropriate. *$p < 0.05$, **$p < 0.01$, ***$p < 0.001$.

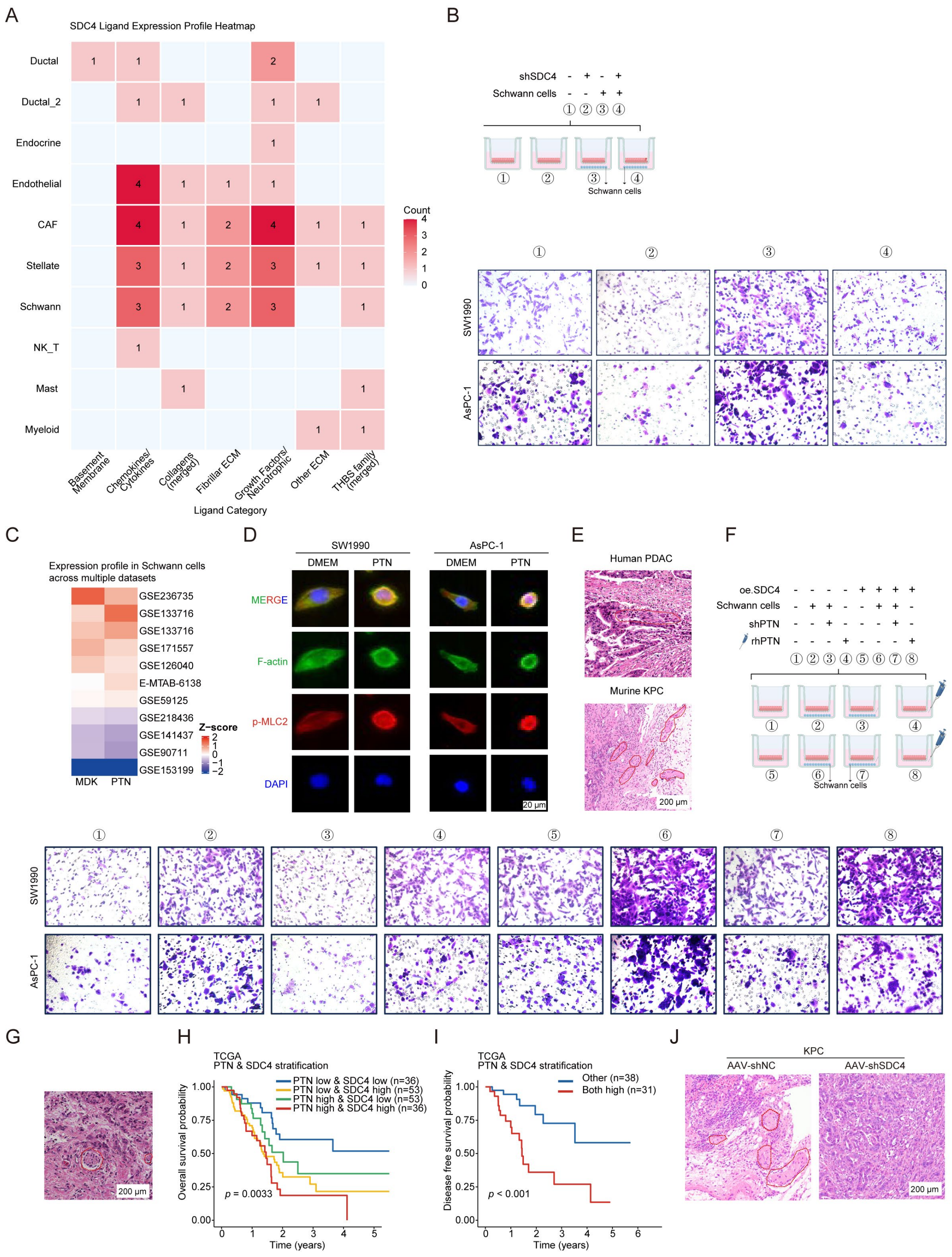

Figure S10 | SDC4–pleiotrophin axis mediates cancer cell–Schwann cell interactions

A) Heatmap showing the number of upregulated SDC4 ligands by category across different cell types. **B)** Transwell invasion assays performed with or without Schwann cells in combination with cancer cell SDC4 knockdown. **C)** Heatmap analysis across multiple bulk RNA-sequencing datasets

of Schwann cells confirming robust and consistent expression of the SDC4 ligands MDK and PTN in Schwann cells. **D)** Effects of PTN treatment (100 ng/ml) on amoeboid transition as demonstrated by immunofluorescence. Scale bars, 20 μm. **E)** Representative hematoxylin and eosin staining of tumor sections showing tumor cell infiltration around nerve structures in human PDAC tumors and murine KPC tumors. Scale bars, 200 μm. **F)** Transwell invasion assays comparing control and SDC4-overexpressing cancer cells under different lower chamber conditions: control treatment alone (without Schwann cells), Schwann cells alone, Schwann cells with PTN knockdown, or 100 ng/ml recombinant PTN protein supplementation alone (without Schwann cells), demonstrating the SDC4-PTN-mediated enhancement of cancer cell–Schwann cell interactions and the resulting increase in cancer cell invasion. **G)** Representative hematoxylin and eosin staining of tumor sections showing tumor cell infiltration around nerve structures in human PDAC tumors. Scale bars, 200 μm. **H-I)** Validation in the TCGA-PAAD dataset showing that dual-high PTN and SDC4 expression predicts worse overall survival **(H)** and disease-free survival **(I)** than other groups. **J)** Representative hematoxylin and eosin staining of tumor sections showing tumor cell infiltration around nerve structures in KPC mice. Scale bars, 200 μm. Survival curves were analyzed using the log-rank test.
